# Protein supplementation during an energy-restricted diet induces visceral fat loss and gut microbiota amino acid metabolism activation: a randomized trial

**DOI:** 10.1038/s41598-021-94916-9

**Published:** 2021-08-02

**Authors:** Pierre Bel Lassen, Eugeni Belda, Edi Prifti, Maria Carlota Dao, Florian Specque, Corneliu Henegar, Laure Rinaldi, Xuedan Wang, Sean P. Kennedy, Jean-Daniel Zucker, Wim Calame, Benoît Lamarche, Sandrine P. Claus, Karine Clément

**Affiliations:** 1 Sorbonne Université, Inserm, Nutrition and Obesity: Systemic Approaches Research Unit, NutriOmics, Sorbonne Université, 91 boulevard de l’Hôpital, 75013 Paris, France; 2 Assistance Publique Hôpitaux de Paris, Nutrition Department, Pitié Salpêtrière Hospital, Assistance Publique Hôpitaux de Paris, 75013 Paris, France; 3 Integrative Phenomics, Paris, France; 4 Sorbonne Université, IRD, UMMISCO, Unité de Modélisation Mathématique et Informatique des Systèmes Complexes, Sorbonne Université, 93143 Bondy, France; 5 YSOPIA Bioscience, 17 place de la Bourse, 33076 Bordeaux, France; 6 Department of Food and Nutritional Sciences, School of Chemistry, Food and Pharmacy, The University of Reading, Reading, RG6 6AP UK; 7 CNRS, Institut Pasteur, Department of Computational Biology, USR 3756 , CNRS, 75015 Paris, France; 8 StatistiCal BV, Strandwal 148, 2241 MN Wassenaar, The Netherlands; 9 Laval University, Institute On Nutrition and Functional Foods, Laval University, Québec, QC G1V 0A6 Canada

**Keywords:** Metabolic syndrome, Obesity, Microbiota, Metagenomics, Microbiome, Randomized controlled trials

## Abstract

Interactions between diet and gut microbiota are critical regulators of energy metabolism. The effects of fibre intake have been deeply studied but little is known about the impact of proteins. Here, we investigated the effects of high protein supplementation (Investigational Product, IP) in a double blind, randomised placebo-controled intervention study (NCT01755104) where 107 participants received the IP or an isocaloric normoproteic comparator (CP) alongside a mild caloric restriction. Gut microbiota profiles were explored in a patient subset (n = 53) using shotgun metagenomic sequencing. Visceral fat decreased in both groups (IP group: − 20.8 ± 23.2 cm^2^; CP group: − 14.5 ± 24.3 cm^2^) with a greater reduction (*p* < 0.05) with the IP supplementation in the Per Protocol population. Microbial diversity increased in individuals with a baseline low gene count (*p* < 0.05). The decrease in weight, fat mass and visceral fat mass significantly correlated with the increase in microbial diversity (*p* < 0.05). Protein supplementation had little effects on bacteria composition but major differences were seen at functional level. Protein supplementation stimulated bacterial amino acid metabolism (90% amino-acid synthesis functions enriched with IP versus 13% in CP group (*p* < 0.01)). Protein supplementation alongside a mild energy restriction induces visceral fat mass loss and an activation of gut microbiota amino-acid metabolism.

*Clinical trial registration*: NCT01755104 (24/12/2012). https://clinicaltrials.gov/ct2/show/record/NCT01755104?term=NCT01755104&draw=2&rank=1.

## Introduction

Obesity is a pandemic disease affecting today an alarming 650 millions adults worldwide and representing the main risk factor for developing chronic metabolic disorders such as type 2 diabetes and other cardiometabolic disorders. Obesity is defined in the western population by an elevated body mass index (BMI = body weight (kg)/[height (m)]^2^) > 30 kg/m^2^. However, there is increasing evidence that adiposity is a better predictor of developing cardiometabolic diseases^1^. One explanation is that the adipose tissue, and particularly visceral fat, is an endocrine organ secreting hormones that regulate appetite and energy metabolism and contributes to systemic low grade inflammation in obese individuals^2^. Consistently, it has been recently documented that visceral obesity and insulin resistance increase the risk of cardiovascular diseases^3^ and type 2 diabetes^4^. Hence, there is increasing interest in identifying factors that control the expansion of visceral fat mass in the hope that acting on these components will help limit the development of obesity-related metabolic co-morbidities.

Fat mass development is strongly correlated with total body weight but currently available strategies to induce weight loss (i.e. hypocaloric diets, increased physical activity, drug-induced reduction of appetite and bariatric surgery) have shown no specific effect on visceral fat mass^5^.

Recent studies identified that gut microbiota are an important factor influencing visceral fat mass independently of diet^6^ and some specific microbial metabolic pathways are associated with visceral fat^7^.This may explain the contrasting findings about the links between dietary macronutrient intake and visceral fat mass. For example, while Le Roy et al. reported that increasing dietary protein intake is associated with higher visceral fat mass^6^, others have reported the opposite^8,9^. The protein composition may also be an important element to consider that may be responsible for diverging results in these studies. This has been well illustrated by a Japanese study comparing milk-derived to soy-derived protein formula where only the milk-derived proteins induced significant visceral fat mass reduction^10^. Most dietary proteins are digested in the upper gastrointestinal tract but low-digestibility protein intake leads to undigested peptides reaching the colon where they can be metabolised by gut bacteria^11^. In the distal intestine, they contribute to the overall production of colonic short-chain fatty acids and there is accumulating evidence of protein-derived metabolites influencing host metabolism^12–16^. Hence, gut microbiota protein metabolism may constitute a key pathophysiological link between obesity, fat mass development and its metabolic complications^17,18^. Previous studies investigating the effects of high protein diets on gut bacteria community composition have reported increased abundance of bile-tolerant microorganisms (e.g. *Alistipes, Bilophila and Bacteroides*) and decreased levels of Firmicutes that metabolise dietary plant polysaccharides (e.g. roseburia*, Eubacterium rectale,* bifidobacteria *and Ruminococcus bromii*)^19–22^. A recent study also reported an increase in *Akkermansia spp.* during a high protein dietary intervention^23^*.*

Therefore, in order to investigate the impact of dietary proteins on visceral fat mass reduction and on the gut microbial ecosystem at both taxonomic and functional levels, we analysed data from a 12-week-long randomized double-blind placebo-controlled energy restriction intervention study in 107 overweight/obese individuals with metabolic syndrome supplemented with high protein formula versus an isocaloric comparative product.

Our hypothesis was that the investigational product (IP) containing a mixture of milk-derived proteins would induce fat mass reduction better than the isocaloric normoproteic comparator containing pea-derived proteins (CP). We also hypothesised that high protein intake would modulate gut microbial functions and questioned whether the protein source would induce a specific functional shift. We observed lower visceral fat mass after 12 weeks of treatment in the IP group. At the gut microbial community level, we observed a modest impact of protein supplementation but a strong functional shift was noted with milk-derived protein supplementation.

## Subjects and methods

### Patient population

This double-blind controlled, multicentre, interventional, randomized in parallel groups (1:1), clinical study was performed in France (Pitié-Salpêtrière hospital, Center of Research of Clinical Nutrition, Institut de Cardiométabolisme et de Nutrition, Paris) and in Canada (Institute on Nutrition and Functional Foods from Laval University, Québec). Patients aged between 18 and 65 years, overweight or obese (body mass index (BMI) ≥ 25 kg/m^2^ and < 40 kg/m^2^) with metabolic syndrome^24^, were selected for inclusion. A complete list of exclusion criteria and study details are provided in supplemental material. Briefly, patients were expected to be free from any known inflammatory disorder, not diabetic and not treated within the last 3 months with drugs affecting visceral fat mass. Antibiotic exposure within the last month, regular intake of food supplements known to affect body weight, satiety or appetite and probiotics were prohibited. Any form of hypocaloric diet or specific diet such as vegetarian or vegan within the last 6 months were also exclusion criteria. The study was registered at Clinicaltrials.gov as NCT01755104 (24/12/2012). Informed consent was obtained form all participants before initiation of any study-related procedure. Research was performed in accordance to the Declaration of Helsinki.

### Dietary intervention and product allocation

A diagram of the study design is available in Supplemental Figure S1. Eligible subjects were randomized to receive a daily supplement of two sachets per day of the high protein investigational product (IP) or the comparator product (CP). IP was a high protein powder preparation containing 34 g of protein, 2 g of fat and 6 g of carbohydrates (i.e. 75%, 12% and 13% of total energy content, respectively) per sachet. Protein sources for the IP were composed of a mixture of milk protein fractions and free amino acids (patent reference: US 20140287057 A1). CP was an isocaloric mixture containing only 7.3 g of protein, 7.6 g of fat and 24.5 g of carbohydrates designed to not alter the overall balance of a conventional diet (i.e. 15% protein, 35% fat, 50% carbohydrate). Protein sources for the CP were composed of hydrolysed pea proteins and calcium caseinate in equal proportion. Both powders were manufactured by ProDietic (France) and were reconstituted in 250 mL of water and taken twice a day as morning and afternoon snacks. A detailed nutritional composition of the IP and CP is given in Supplemental Table S1. The IP and the CP were administered along with a balanced (50% carbohydrates, 35% fat and 15% proteins) diet with a moderate caloric restriction defined by a reduction of 600 kcal from the estimated daily caloric needs, for 12 weeks and for another 4-week diet-free, maintenance period. Daily caloric needs were estimated by a registered dietician, as product of the energy expenditure (REE) following the Harris and Benedict formula multiplied by a coefficient of 1.3 (for a sedentary lifestyle). The daily portion provided by the IP or the CP (360 kcal/day), was included in the calculation of the total energy intake. Subjects in both study groups were instructed to maintain their usual physical activity habits during the study period. Dietary advice was given to subjects in the investigational centre at the inclusion visit, follow-up visit 1 and follow-up visit 2. A phone call, initiated by the dietitian, was planned every week for the whole period of the study. Before and at the end of the intervention, food intake was quantitatively measured using population-specific validated food frequency questionnaire completed by both French and Canadian subjects online^25^.

### Study endpoints

The primary endpoint was a change in the abdominal visceral fat area (VFA) measured in cm^2^ from baseline to Week 12 (end of intervention). Abdominal VFA was measured 5 cm above L4–L5 intervertebral disc using a computed tomography (CT) scan at both centres. Reading of the CT scan was performed centrally (Philips NCTC 965 Software), for the complete set of subjects, by a single reader (radiologist). Secondary endpoints included changes in body composition, cardiometabolic risk factors, inflammatory parameters and gut microbiota composition. Compliance was assessed by recording the number of delivered and returned sachets, including empty sachets. Safety was assessed based on the reporting of adverse events monitored from the time that the subjects gave informed consent to the end of the study.

### Fecal microbiota analysis

Participants to this study in the French centre had faecal sampling before and after the intervention.

#### Extraction, sequencing and analysis of faecal genomic DNA

Detailed information about extraction, sequencing and analysis of faecal microbiota is provided in the supplementary methods. Briefly, DNA sequencing data were generated using Illumina HiSeq2500. Normalisation and downsizing were performed and the abundance of MGS (metagenomic species) > 500 genes was computed as described^26^. Alpha-diversity was measured in two ways: gene richness i.e. the average number of genes (meaning at least one read mapped) per sample and MGS richness i.e. the MGS present in each sample. Enterotyping of the cohort was performed following the Dirichlet Multinomial Mixture (DMM) method^27^ using MGS abundance matrix of the entire cohort collapsed to genus level. Functional characteristics of the metagenomes were assessed for each sample by collapsing gene abundance into KEGG modules as described^28^ based on KEGG functional mappings of the IGC gene catalog (PMID: 24997786; PMID: 10592173). To complete this functional module matrix (where amino-acid degradation modules are not fully represented), gut metabolic modules as described by Viera-Silva et al.^29^ were computed for each sample using omixerRPM v0.2.3 R package^30^. Using functional annotations of the 9.9 M genes catalogue, prevalence matrices (presence/absence) of functional annotation per MGS were computed allowing the bioinformatic constitution of amino-acid synthesis and degradation functional groups of MGS.

### In vitro batch fermentation

Detailed information on in vitro anaerobic batch fermentation protocols and faecal microbiota analyses are provided in supplementary methods. Briefly, pre-digested protein mix (0.35 g) was added to the sterile vessels with basal nutrient medium prior to inoculation with 2 mL of faecal inocula from 6 selected donors (3 lean and 3 obese, matched for sex and age). Samples were collected at baseline (T0) and after 48 h fermentation (T48). Following faecal DNA library preparation, metagenomic sequencing was performed with Illumina HiSeq. Microbiota characteristics were assessed using the same process as for clinical study samples.

### Statistical methods

Sample size calculation is detailed in supplementary methods. The Full Analysis Set (FAS) population consisted of all randomized subjects who consumed at least one sachet of the study IP. The Per Protocol (PP) population included all subjects who completed the study without any major protocol deviation (as detailed in supplementary methods). Since microbiome samples were only available at T0 and T12 weeks, in the present manuscript we focused entirely on results at these two time points and data from T16 weeks were not considered.

### Statistical analysis of clinical data

Absolute changes of the primary and secondary outcomes, based on FAS population and measured at Week 12, were compared between the two groups using stepwise General Estimating Equations (GEE) analysis in a repeated fashion adjusted for centre (French/Canadian), age, sex, BMI, treatment, time, interaction between treatment and time, and starting value. Additional analyses for the primary endpoint were conducted using the PP population. Quantitative variables were described using mean, standard deviation, median, minimum and maximum. Qualitative variables were described using frequency and percentages. To investigate whether consumption of IP decreased the visceral fat mass to a greater extent than CP, a dummy stepwise multifactorial (GEE) model was applied in a repeated (participant) fashion. The multifactorial model was stratified by centre and adjusted for age, sex, BMI, treatment, time, interaction term of treatment and time, start value. Change in visceral fat mass per person between week 6, week 12 and week 0 was used as dependent parameter. Goodness of Fit of the model was evaluated using a Wald Chi-square statistic. Throughout the study a *p* value below 0.05 was considered to detect a statistically relevant difference applying two-sided evaluation. Outlier analysis was conducted via the Grubbs test (two-sided with α-level of 0.05). Patients with inconsistent dietary declarations i.e. energy intake < 0.5 * Basal metabolic rate (BMR; estimated using Harris and Benedict formula) or energy intake > 3 * BMR were excluded from dietary intake statistical analysis.

### Statistical analysis of microbiome data

Microbiome dynamic changes were calculated as the log-ratio of abundance at T12 versus T0 at the different taxonomic levels after filtering for > 20% prevalence at baseline. For representation of changes over time, cliff delta effect of time was shown for MGS and 16S calculated genera (nonparametric distribution). For other microbiota features, log fold change (i.e. log (T12/T0)) is shown. Microbiota changes were analysed in linear mixed models using fixed effect of time, adjusted for baseline age, sex and baseline for pooled analysis and interaction of time with intervention with same adjustments for between groups analysis. Beta-diversity was computed using Bray–Curtis distance with vegdist function of the vegan R package (v2.5–6)^31^ from MGS abundance matrix collapsed at genus level. Principal Coordinate Analysis (PCoA) of Bray–Curtis beta-diversity matrix was carried out with the cmdscale function of vegan R package. When relevant, adjustment for multiple comparison was performed using the False Discovery Rate method (FDR). For FDR adjusted analysis, the statistical significance threshold was set to 0.1. Statistical analyses and conception of figures were carried out using R version 3.3.2, R Core Team (2019), https://www.R-project.org/.

### Ethics approval and consent to participate

The study was registered at Clinicaltrials.gov as NCT01755104. The study protocol was reviewed and approved in local ethical committee For France, local ethical committee was *CPP IDF 1; 00008522* (study approval reference: 2012-sept-13025) and for Canada, local comitee was the *comité d'éthique de la Recherche de l'IUCPQ* (study approval reference: 20922). Informed consent was obtained from each subject before initiation of any study-related procedure.

## Results

### Results of the main clinical trial analyzing the whole cohort of both French and Canadian patients: effects of protein supplementation on body composition and metabolic parameters

#### Baseline characteristics in IP and CP groups

A total of 107 subjects were included in the Full Analysis Set (FAS) population and 99 in the per protocol (PP) population (Study flow chart, Figure S2). Overall baseline characteristics were similar in both groups (Table 1, Table S2).

**Table 1:** Baseline subject characteristics for the FAS population.

	IP (n = 54)	CP (n = 53)	*p* value
**Age (years)**	47.7 (10.2)	48.1 (11.3)	0.64
Male (N,%)	24 (44.4)	20 (37.7)	
Female (N, %)	30 (55.6)	33 (62.3)	
BMI (kg/m^2^)	32.4 (3.8)	32.2 (3.7)	0.73
Waist circumference (cm)	104.5 (9.4)	103.4 (8.3)	0.54
Waist/Hip circumference ratio	1.0 (0.1)	0.9 (0.1)	0.56
Visceral fat area (mm^2^)*	213.0 (87.7)	181.6 (59.2)	0.10
Fasting plasma glucose (mmol/L)	5.53 (0.71)	5.62 (0.63)	0.49
HDL (mmol/L)	1.2 (0.4)	1.2 (0.4)	0.71
LDL cholesterol (mmol/L)	3.2 (0.8)	3.4 (0.9)	0.62
Triglycerides (mmol/L)	1.6 (0.9)	1.7 (0.8)	0.58
CRP (mg/L)	7.31 (21.54)	4.35 (6.21)	0.34
TNFalpha (pg/mL)	1.85 (1.56)	1.40 (0.66)	0.05*
IL-6 (pg/mL)	2.68 (5.11)	1.72 (0.89)	0.18

Mean +/− SD; BMI: Body mass index; *p < 0.05 between IP and CP; Distribution in men and women was equivalent in both groups (*p* = 0.48).

#### Compliance and safety analysis

Median compliance was high, reaching 96.5% at week 12 and was similar in each group. Over 96% of subjects in each group reported good tolerance of the IP and CP products. Detailed information about adverse events is provided in Supplementary Table S3 and S4. There were no signs of kidney toxicity with no significant changes in creatininemia in neither IP nor CP groups (Table S5).

#### Dietary intake

For 9 patients dietary data were missing and 4 were excluded because of inconsistent declarations. Dietary intervention led to an energy restriction that was similar in IP and CP groups, while maintaining constant fibre intake (Table 2). At the end of the intervention (T12), although carbohydrate intake by design also differed, the main difference between the 2 groups was protein intake.

**Table 2:** Description of the nutritional intake before and after the intervention.

	Baseline (T0)			T12 weeks: diet only			T12 weeks: diet + daily supplement		
	IP n = 48	CP n = 46	*p*	IP n = 48	CP n = 46	*p*	IP n = 48	CP n = 46	*p*
Energy intake (kcal)	2436 (899)	2255 (872)	0.32	1414 (483)	1414 (518)	0.99	1770 (496)	1790 (530)	0.85
Fat (g)	99.9 (43.1)	85.4 (40.1)	0.01	51.2 (22.3)	49.1 (24.6)	0.66	56.9 (23.1)	63.7 (25.0)	0.17
Fat (%)	36.3 (5.30)	33.6 (5.56)	0.02	32.1 (7.33)	30.3 (5.78)	0.17	28.2 (5.91)	31.5 (4.39)	0.003
Carbohydrates (g)	271 (101)	269 (102)	0.92	166 (61.6)	168 (63.8)	0.85	176 (62.0)	214 (65.0)	0.005
Carbohydrates (%)	44.9 (5.90)	48.1 (7.18)	0.02	46.6 (8.60)	47.9 (6.61)	0.42	39.3 (6.93)	48.0 (5.13)	< 0.001
Protein (g)	108 (45.7)	97.2 (42.3)	0.25	68.8 (24.0)	73.3 (23.4)	0.36	134 (24.8)	87.5 (23.9)	< 0.001
Protein (%)	17.7 (3.77)	17.2 (3.12)	0.43	20.0 (5.81)	21.2 (3.55)	0.23	31.5 (5.50)	19.8 (2.62)	< 0.001
Fibre (g)	23.5 (10.8)	23.7 (10.3)	0.91	17.9 (8.14)	18.2 (6.68)	0.83	21.3 (10.4)	18.1 (6.5)	0.09

Data is expressed as mean (SD) for continuous variables. Macro-nutrient intake is expressed as g/day or percent of total energy intake when specified. *P* values of Student t test.

### Effect of protein supplementation on body composition and cardiometabolic risks

#### Protein supplementation induces changes in visceral fat and body composition

**Figure 1:**
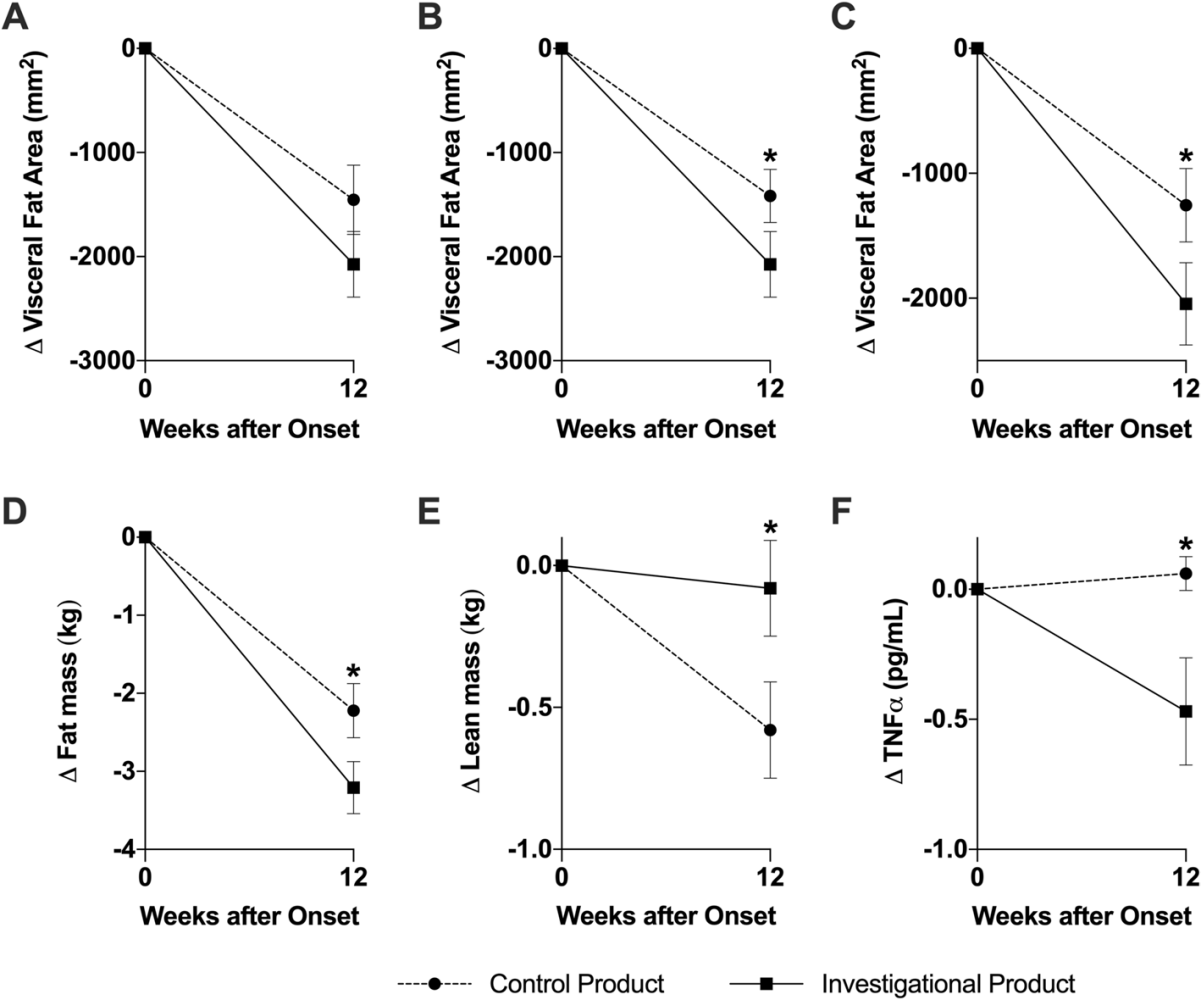
Investigational Product improved fat mass, maintained lean mass and reduced inflammation at week 12 post intervention. Changes in visceral fat area as established via CT scan in the (**A**) FAS population, (**B**) FAS without outliers and (**C**) PP population. Changes in fat mass (**D**) and lean mass (**E**) as established via DEXA scan and in TNFα (F) in FAS without outliers. Mean (SEM) for at least n = 51. *p < 0.05 (GEE model) Key: Circle is Control Product; Square is Investigational Product.

Mean visceral fat area (VFA) in the FAS population decreased significantly from baseline to Week 12 in both IP (− 20.7 ± 23.2 cm^2^ i.e. − 9.7% from baseline, *p* < 0.0001) and CP (− 14.5 ± 24.3 cm^2^ i.e. − 8.0% from baseline, *p* < 0.0001) groups. Elimination of 2 outliers (determined via Grubbs test) revealed a significant difference in the absolute reduction of visceral fat area between the IP and CP group (− 20.8 vs. − 14.2 mm^2^ resp, *p* < 0.05). The absolute reduction of visceral fat area from baseline to Week 12 was not significantly higher in the IP compared to CP groups (*p* = 0.09) in the FAS population but was significantly higher in the PP population (− 20.5 vs. − 12.6 cm^2^ resp., *p* < 0.05) (Fig. 1). In the FAS population, the between group adjusted difference was significant for total fat area (*p* < 0.05), Subcutaneous Fat Area (*p* < 0.05) and Fat-free mass (*p* < 0.05) in favour of the IP group (Table 3). There was no absolute change in fat-free mass (i.e. lean mass) from baseline to Week 12 in the IP group, whereas a significant decrease (*p* < 0.001) was observed in the CP group (*p* < 0.01 between groups).

**Table 3:** Between-group differences in absolute changes in body composition from baseline to Week 12 in the FAS Population (N = 107).

	IP (N = 54)		CP (N = 53)		IP versus CP (N = 107)	
	Mean absolute change (SD)	*p* value	Mean absolute change (SD)	*p* value	Mean absolute change	*p* value
Body weight (kg)	− 3.56 (3.12)	< 0.001	− 3.07 (3.09)	< 0.001	0.49	0.701
Total fat mass (kg)	− 3.21(2.47)	< 0.001	− 2.22 (2.53)	< 0.001	0.99	0.004
Visceral Fat Area (cm^2^)	− 20.7 (23.2)	< 0.001	− 14.5 (24.3)	< 0.001	− 6.2	0.088
Total Fat Area (cm^2^)	− 46.6 (43.8)	< 0.001	− 30.8 (48.3)	< 0.001	− 15.8	0.010
Subcutaneous Fat Area (cm^2^)	− 25.9 (32.3)	< 0.001	− 16.3 (32.5)	< 0.001	− 9.6	0.029
Fat-free mass (kg)	− 0.08 (1.24)	0.644	− 0.58 (1.24)	0.001	0.50	0.003
Fasting glucose (mmol/L)	− 0.17 (0.45)	< 0.001	− 0.3 (0.49)	< 0.001	0.47	0.249
HDL cholesterol (mmol/L)	− 0.02 (0.19)	0.468	0.03 (0.14)	0.115	0.05	0.301
LDL cholesterol (mmol/l)	− 0.3 (0.6)	< 0.01	− 0.2 (0.7)	< 0.01	− 0.1	ns
Triglycerides (mmol/L)	− 0.28 (0.61)	< 0.002	− 0.37 (0.55)	< 0.001	0.09	0.296
CRP (mg/L)	− 3.4 (19.8)	0.205	1.0 (9.9)	0.457	4.4	0.049
TNF alpha (pg/mL)	− 0.47 (1.57)	0.023	0.06 (0.48)	0.401	0.53	0.002
IL-6 (pg/ml)	− 0.83 (5.03)	0.223	0.17 (1.46)	0.383	1.00	0.151

Mean values (standard deviation); Between group difference denotes IP effect versus CP.

#### Changes in cardiometabolic risk factors and inflammatory markers

Systolic and diastolic blood pressures, BMI, waist circumference, fasting blood glucose, total cholesterol, LDL cholesterol and triglycerides levels significantly decreased from baseline to 12 weeks (*p* < 0.001) without significant between group effects (IP vs. CP) (Table S5). CRP (C-reactive Protein) and TNF alpha, two inflammatory markers commonly associated with low grade inflammation in obesity, were significantly lower in the IP group versus CP group (*p* < 0.05 and *p* < 0.01 respectively) (Table 3). On the other hand, the decrease in HbA1c was significantly more substantial in CP group versus IP (Table S5).

### Results of the ancillary study focusing on the subcohort of French patients: effects of protein supplementation on the gut microbiota

#### Effect of protein supplementation on the gut microbiota composition

Out of the 53 participants with microbiota sampling (i.e. the French participants), 2 were excluded because of an inter-current antibiotic treatment (Figure S3).

#### Increase in gut microbiota richness associates with visceral fat mass loss

**Figure 2:**
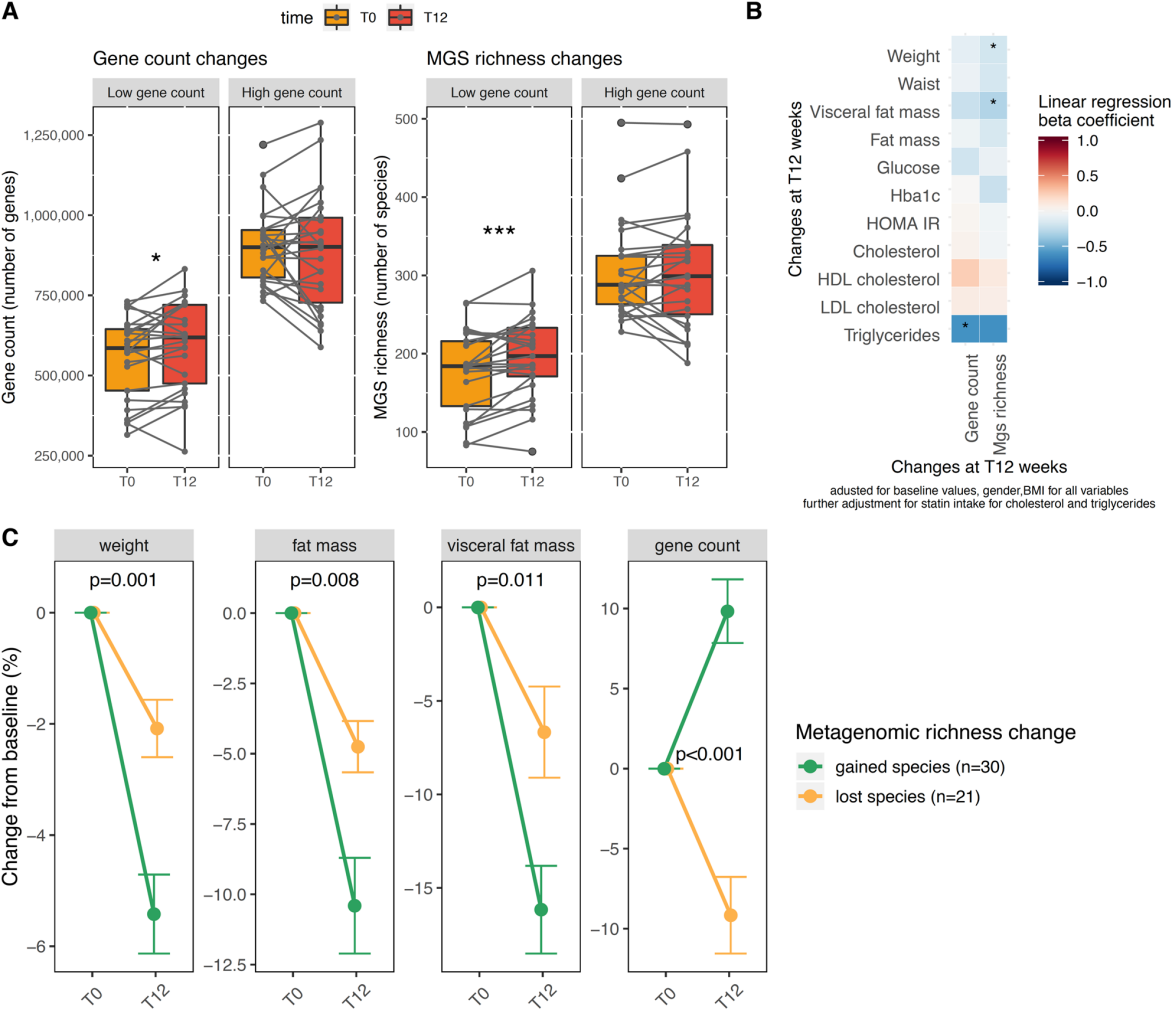
Evolution of metagenomic richness 12 weeks after dietary intervention. (**A**) Evolution of gene count (number of genes) and metagenomic richness (number of MGS) depending on baseline gene richness status The fixed effect of time (the intervention) was analysed in a mixed linear model with patients as random effects. *p < 0.05, **p < 0.001. (**B**) Evolution of richness associations with the clinical evolution. Heatmap of standardized beta coefficient from linear regression. Model is adjusted for baseline values + baseline BMI and sex. For cholesterol and triglycerides, the model is also adjusted for baseline statin intake. (**C**) Evolution of weight, fat mass, visceral fat mass and gene count depending on richness response. Change from baseline is (T12–T0/)T0 * 100. Gained species are individuals who increased their MGS richness. Lost species are individuals who decreased their metagenomic (MGS) richness. P values of the effect of MGS richness change in a linear regression model adjusted for baseline value, sex and BMI. Points are mean, bars are SEM. Figure conceived using R version 3.3.2, R Core Team (2019), https://www.R-project.org/.

Overall, the dietary intervention was not associated with changes in microbial diversity neither in number of genes or number of species and there was no difference between groups. However, individual trajectories were highly heterogeneous and consistently with previous observations^19^, the change in diversity after the intervention depended on the baseline diversity status. Independently of IP or CP supplementation, individuals with a baseline low gene count, had a significant increase in diversity (gene count: 6.6 ± 13.6% vs. − 2.3 ± 13.9% for low vs. high baseline gene count individuals; *p* = 0.015; metagenomic richness: 11.6 ± 17.8% vs. − 0.4% ± 12.3% for low vs. high baseline gene count individuals; *p* = 0.0009) (Fig. 2A). Interestingly, individuals that increased their number of metagenomic species during the intervention were those who showed the greatest weight, fat mass and visceral fat mass loss (Fig. 2B,C).

#### Protein supplementation had modest impact on microbial diversity and composition

**Figure 3:**
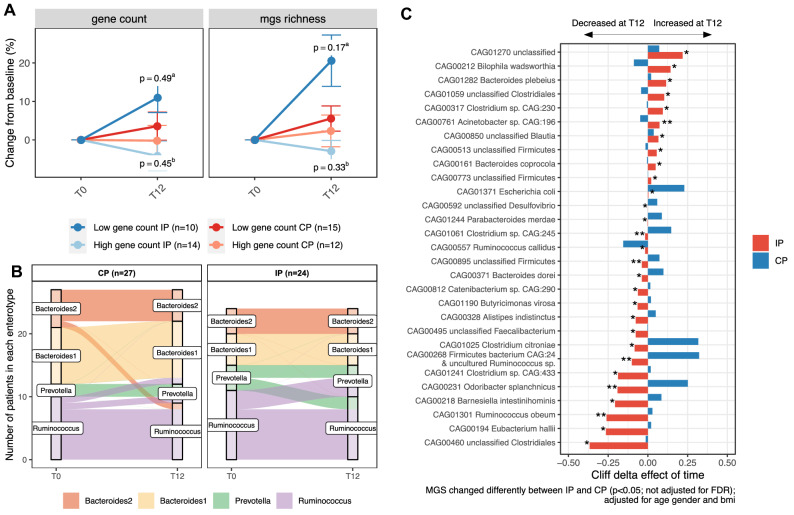
Effects of protein supplementation on gut microbiome composition (IP vs. CP). (**A**) Evolution (relative change at T12) of gene count and MGS richness (number of present species) in investigational product group (IP, red colours) and Comparator group (CP, blue colours) depending on baseline metagenomic richness. LGC: low gene count at baseline (light colours). HGC: high gene count at baseline. p value of the effect intervention interaction with time in LGC patients (a) and HGC patients (b); (**B**) Alluvial plot showing the evolution of enterotype between T0 and T12 in CP group (left panel) and IP group (right panel); (**C**) Untargeted analysis of the effects of IP on metagenomic species (MGS) abundance changes with the intervention. MGS shown are the ones with a significant interaction of time with intervention in a mixed linear model with patients as random effects adjusted for baseline age, sex and BMI (*p* < 0.05 without adjustment for multiple comparisons). Bars represent the cliff delta effect of time (T12 vs T0) on MGS abundance in each group. *p < 0.05; **p < 0.01; ***p < 0.001 No significant differences resist to adjustment for multiple comparisons. IP: investigational product (high protein); CP: comparator product. Figure conceived using R version 3.3.2, R Core Team (2019), https://www.R-project.org/.

Calorie restriction-induced weight loss was similar in IP and CP and the main difference between the two intervention groups in terms of dietary intake changes was protein intake (Table 2). Despite this major low-digestibility protein supplementation, no significant changes were observed in alpha-diversity in IP versus CP group (Fig. 3A). Beta-diversity did not differ between the two groups at baseline (permanova *p* = 0.47; R^2^ = 0.018) nor at the end of intervention (T12) (*p* = 0.34 R^2^ = 0.021). There was no effect of intervention on beta-diversity in the CP group (*p* = 0.629; R^2^ = 0.014) nor in the IP group (*p* = 0.93; R^2^ = 0.007) (Figure S4A–D).

After 12 weeks, 8 patients switched their enterotype (15%). The proportion of subjects that changed their enterotype was lower when the baseline enterotype was *Bacteroides* (n = 1, 2.0% vs. n = 7, 13.7%; *p* value = 0.024). The proportion of enterotype change was not different between the 2 groups (9.8% in IP group vs. 5.8% in CP group, *p* = 0.34) (Fig. 3B).

We next investigated whether microbiome changes induced by higher protein supplementation with IP could be observed at lower taxonomic levels. The 16S targeted analysis of known taxa associated with metabolic health revealed modest, but interesting, modulations (Figure S5). *Akkermansia spp.* tended to increase with energy restriction (in both groups) while bifidobacteria tended to decrease. *Christensenella spp.* and *Lactobacillus sp.* tended to increase in the IP group, while *Turicibacter spp.* tended to be boosted in the CP group (non-significant trends). Regarding untargeted shotgun metagenomics results, some MGS were increased in the IP versus CP groups, such as *Bilophila wadsworthia* but none of these changes were significant after adjusting for multiple comparisons (False discovery rate < 0.1) (Fig. 3C).

#### IP supplementation induces amino acid metabolism functional changes in the gut microbiota

**Figure 4:**
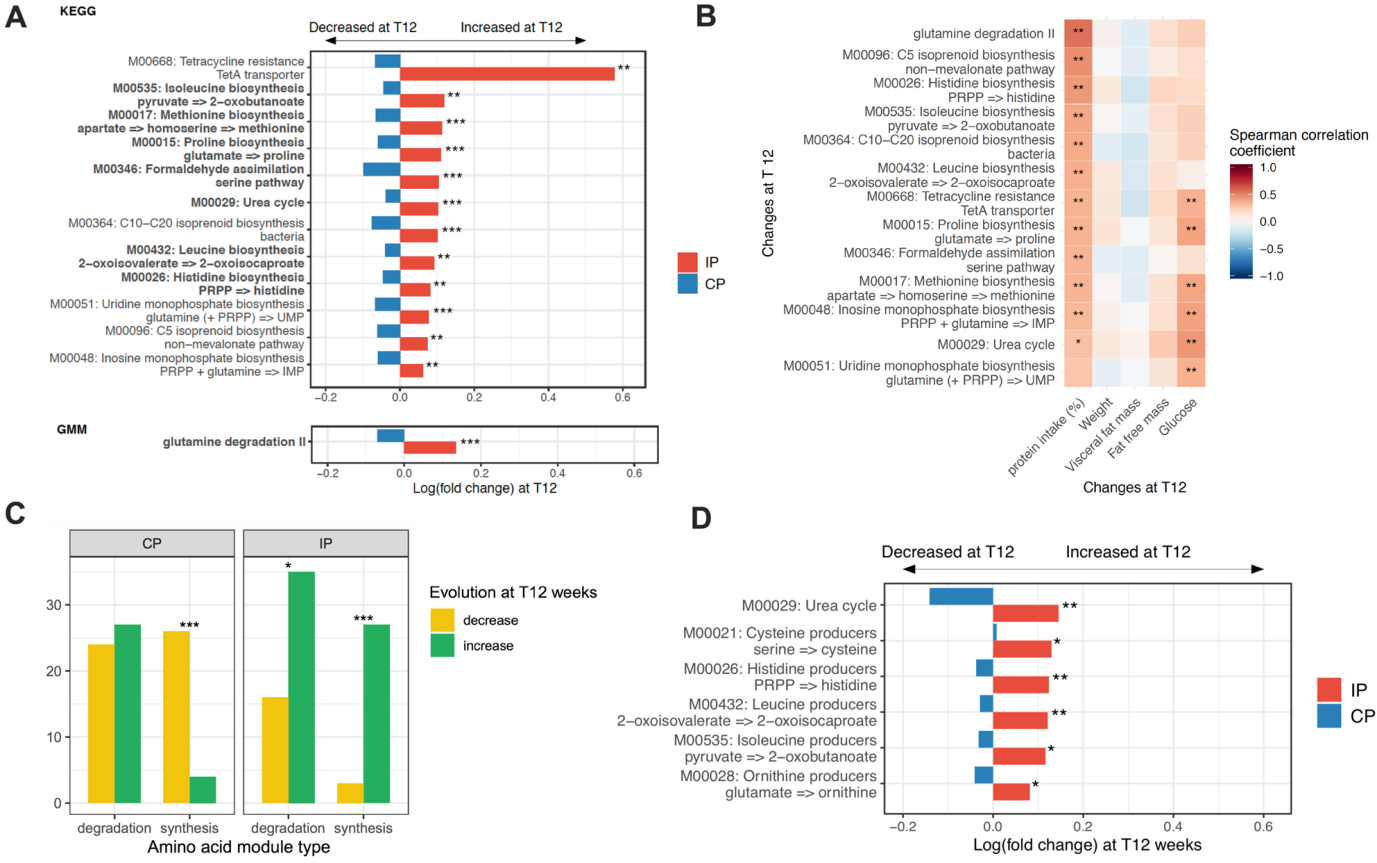
Effects of protein supplementation on gut microbiota function. (**A**) Effects of IP on functional modules from KEGG and GMM databases. Shown modules are those which changed differently (FDR < 0.1) between the two groups (IP vs. CP) adjusted for baseline BMI, age and sex. Bars represent log(fold change) at T12 of modules abundances in each group (IP vs. CP). Modules in bold: amino-acid metabolism modules. *p < 0.05; **p < 0.01; ***p < 0.001. (**B**) Heatmap of spearman correlations between functional modules fold changes and bio-clinical relative changes after the intervention (T12-T0/T0); *p < 0.05; **q < 0.05 (adjusted for multiple comparison, FDR method). (**C**) Evolution of the amino acid metabolism (degradation and synthesis) functional modules (KEGG and GMM) with the intervention. Increase is defined by a mean fold change > 0 and decrease by a mean fold change < 0 for each module. The observed proportion of increased module was compared to a theoretical value of 0.5 with a binomial test. *p < 0.05; **p < 0.01; ***p < 0.001 (adjusted for multiple comparisons, FDR method). (**D**) Effects of IP on amino acid metabolism functional groups of metagenomic species. Shown functional groups are those which changed differently (*p* < 0.05) between the two groups (IP vs. CP) adjusted for baseline BMI, age and sex. Bars represent log(fold change) at T12 of modules abundances in each group (IP vs. CP). *p < 0.05; **p < 0.01; ***p < 0.001. IP: investigational product (high protein); CP: comparator product. Figure conceived using R version 3.3.2, R Core Team (2019), https://www.R-project.org/.

Twelve KEGG functional modules were induced by the IP with a significant difference compared to the CP group (Fig. 4A) (IP vs. CP adjusted for age, BMI and sex; *p* < 0.001 and FDR < 0.1). Interestingly, most of these modules were involved in amino acid metabolism such as *urea cycle*, amino-acid biosynthesis modules (*histidine, leucine, lysine, proline and methionine biosynthesis*). One amino acid degradation module (glutamine degradation II) was induced by the IP based on the same statistical approach on the Gut Metabolic Modules (GMM) repository (Fig. 4A). Moreover, these functional changes were positively correlated with patients’ individual % protein intake changes from baseline (Fig. 4B) and were not linked with weight loss. Complementing this approach, we then focused on amino-acid metabolism and compared the evolution of amino acid synthesis and degradation modules in IP versus CP groups. Most amino acid synthesis modules were enriched (i.e. mean abundance higher at T12 vs T0) during intervention in the IP group whereas the opposite was observed for the CP group (90.0% for IP group vs. 13.3% in CP group; chi square *p* < 0.001). A majority of amino acid degradation modules were also enriched in the IP group but this did not reach significance compared to the CP group (68.6% in the IP group vs. 52.9% in the CP group; chi square *p* = 0.10) (Fig. 4C). To further explore these functional changes, we projected amino acid metabolism modules from KEGG and GMM databases on metagenomic species (MGS), computing functional groups of bacteria with the capacity to synthesise (i.e. producers) or degrade (i.e. degraders) amino acids. We observed significant increases in amino-acid producers (Cysteine, Threonine, Isoleucine, Leucine, Histidine and Ornithine) induced by IP (vs. CP) with no effect on amino acid degraders (Fig. 4D). However, when comparing the coverage of these functional modules across MGS gene content, we found that the completion of biosynthesis modules was from far higher than degradation modules (Figure S6A), with a large overlap between biosynthesis and degradation phenotypes for different amino acids (Figure S6B).

### Results of the in vitro study: effects of the protein fraction alone on the gut microbiota

#### Protein supplementation induces functional microbiota changes during in vitro bacterial fermentation

To confirm that metagenomic changes observed in the IP group were due to an increased amount of protein reaching the colon, we examined the effects of a direct supplementation of the protein extracted from both CP and IP on microbiota function using an in vitro fermentation approach. We confirmed that exposure to proteins from IP or CP was associated with increased amino acid metabolism in both cases (Figure S7), with a moderate specific effect of IP versus CP (Figure S8A,B). This suggests that the differences observed in the clinical dietary intervention study could partially be due to the low digestibility qualities of the proteins contained in the IP. Furthermore, in this experiment where proteins were the only source of carbon for bacteria, most of the increases observed corresponded to amino acid degradation modules with a less marked effect for synthesis modules (Figure S8C).

## Discussion

In this 12-week long energy-restricted dietary intervention, we observed visceral fat mass loss without lean mass loss in subjects that received the high milk-derived protein investigational product (IP) in the per protocol population. We observed a strong interaction between a gain in individuals’ gut microbiota richness and both body weight and visceral fat mass loss, independently of protein supplementation. Protein supplementation induced significant functional changes on bacterial amino acid metabolism with increases in both amino acid synthesis and degradation, whereas it had a limited impact on the alpha or beta diversity. Since the gut microbiota analyses concerned only half of the population of the study, we cannot conclude whether these microbiota changes underlined the effects of the IP observed on visceral fat mass at the scale of the full cohort.

The importance of the macronutrient composition for efficiency of such moderate weight loss diets is still subject to debate^32^. Herein we show that alongside a mild calorie restriction, a high protein supplementation limits lean mass loss in line with previous reports^8,33–36^. In the per protocol population, we also observed that IP supplementation promoted visceral fat mass loss similarly to previous findings^9,10^. Interestingly here, this was associated with a decrease in low grade inflammation markers.

Recent studies underline the connection between visceral fat mass and the gut microbiota^6,7^. We show here for the first time the link between gut microbiota changes and visceral fat mass.

Gut dysbiosis, defined by a low metagenomic diversity and altered composition, has been associated with moderate to severe obesity and its metabolic complications^19,37,38^. We first confirm here in a distinct human population that a dietary intervention combining energy restriction and protein enrichment improves metagenomic diversity in individuals with a baseline low gene count, in agreement with results from our previous study^19^. We also show that while the individual microbiota response to a dietary intervention is highly heterogeneous, it is nonetheless intricately linked with body composition improvement. Subjects who improved their microbial diversity (net gain of species), lost significantly more weight and visceral fat mass compared to those who experienced a net loss of metagenomic species. Consistent with previous findings, we noted few enterotype changes during this dietary intervention^13^. Interestingly, the *Bacteroides* enterotype, shown to be associated with animal protein consumption, was the most resilient to this high protein dietary intervention. More specifically, the *Bacteroides 2* enterotype, known to be associated with a more severe metabolic phenotype^39^ was not reversed with this moderate weight loss intervention.

The unique design of this study with an iso-caloric, iso-fibre control diet, and the use of both 16S and metagenome sequencing analyses, allowed us to examine the specific effects of high protein intake on the gut microbiome composition and function. The 16S data analysis revealed modest modulations of interesting genera such as *Akkermansia* spp. that tended to increase in both groups due to diet restriction consistently with recent findings^23^ and *Christensenella* spp. that showed a trend for increase in the IP group and which as been previously correlated with an increase in amino acid-derived metabolites in response to high protein intake^40^. Nevertheless, none of these changes were significant. The metagenomic analysis revealed a few changes at the species levels, some of which have already been described in previous studies. *Bilophila wadsworthia*, has been associated with protein intake^20^ and is one of the metagenomic species with the biggest increase in the high protein IP group in our study. Importantly, this species has been recently linked with glucose dysmetabolism^41^. Among the other metagenomic species increased in the IP group, two of them, *Bacteroides coprocola* and *Bacteroides plebeius*, belong to the Bacteroides genus, which has previously been associated with a high protein diet^20^. However, overall, protein supplementation was found to induce only minor modulations in bacterial composition. Although this can be somewhat surprising, it is in line with previous results^40,42^. However, taxonomic changes do not accurately reflect potential functional changes^43^ and the protein sources tested here had a different impact at this level. IP supplementation was associated with a functional switch towards increased amino acid metabolism by bacteria. Glutamine degradation was among the main IP-induced functional changes. Interestingly, glutamine degradation was a key over-represented function in the microbiota of a population with a protein-rich diet or carnivorous mammalians^43,44^. Beside glutamine degradation, most specific changes induced by the IP compared to the CP involved amino acid synthesis functional modules and this was confirmed in complementary analysis focusing on functional groups of MGS. However, we highlighted a potential lack of genomic coverage for enzymatic activities involved in amino acid degradation in metagenomic gene catalogues. This is illustrated by the branched-chain dehydrogenase enzyme complex^45^, whose coverage is very sparse across MGS of the IGC gene catalogue. Indeed, none of the MGS harbour all specific subunits of the enzyme complex or additional steps of the degradation pathway but only the first step^46^ as illustrated in (Figure S9). This lack of coverage for critical enzymes of amino acid catabolism together with incompleteness of MGS assemblies and the potential overlap of some amino acid metabolism function (Figure S6) can influence the quantification of degradation modules in quantitative metagenomic studies. Despite these methodological issues, the increase in amino acid synthesis in IP versus CP group suggests a higher bacterial protein anabolism stimulated by milk-derived proteins, that may result from increased availability of nitrogen in the gut, which may prompt de novo synthesis of amino acids. Another hypothesis is the increased urea and ammonia excretion into the gut that has formerly been associated with a high protein diet^47^. This excess nitrogen in the gut can be utilized by bacteria and may explain the observed enhanced amino-acid metabolism in our study. Supporting this hypothesis, changes in microbiota amino acid metabolism specific to the IP were less pronounced in the in vitro batch fermentation study where these functional changes were also found in the CP group, although to a lower extent. This could be explained by the likely absence of increased urea and ammonia excretion in the in vitro experiment. Also, in the fermentation experiment design, proteins were delivered directly to the faecal samples without prior absorption of amino acids that normally occurs in the small gut in vivo*.* This suggests a different fate for proteins depending on their quality (directly impacted by the protein source) and digestibility, in agreement with previous reports^40,48^.

It is worth noting that limitations frequently affecting other human dietary interventions are also present in our study. The IP group had somewhat lower carbohydrate intake and could explain some of the observed microbiome changes. Nevertheless, protein intake was the main difference between IP and CP groups and, unlike some previous studies, no differences in fibre intake was observed. It is important to note that although it was not significant, there was a trend for increased fiber intake in CP versus IP. Although overall protein intake was more than 50% higher in the IP group, both interventions were characterised by a rather high proportion of proteins in total energy intake (21% in CP vs. 34% in IP group). Regarding the in vitro fermentation study, the conditions were optimized to reproduce the standard digestion process of proteins in the upper gastrointestinal tract before reaching the colonic microbiota. The 48 h batch fermentation therefore mimics the adaptation of the gut microbiota to a sudden exposure to high proteins rather than a chronic exposure to a high protein diet over the 12 weeks of the intervention study. Finally, we do not have access to the profile of fecal amino acids, which would be interesting to measure in further intervention studies to confirm the observed functional switches.

In conclusion, in this dietary intervention study, we show that moderate caloric restriction coupled with a high protein supplement decreased visceral fat mass while maintaining lean mass. We confirmed that a gain in metagenomic diversity is a strong marker of a positive reponse to a dietary intervention in terms of body composition changes and observed for the first time a link between microbiota diversity improvement and visceral fat mass loss. Diet enrichment in proteins had only small effects on bacterial community composition but led to significant functional changes in the gut microbiota with an activation of amino acid metabolism. Deeper studies of the impact of different diets on the gut microbiota composition and function could be key to better understand the heterogeneous responses commonly observed after dietary interventions.

## Supplementary Information

Supplementary Information.

## Data Availability

Data described in the manuscript, code book, and analytic code will be made available upon request pending. Sequencing data have been deposited in the European Bioinformatics Institute (EBI) European Nucleotide Archive (ENA) under accession number PRJEB35524.

## Abbreviations

BMI: Body Mass Index
BMR: Basal Metabolic Rate
CP: Comparator Product
CRP: C-reactive Protein
CT scan: Computerized Tomography scan
DNA: Deoxyribonucleic Acid
FAS: Full Analysis Set
FDR: False Discovery Rate
GMM: Gut Metabolic Modules
HDL: High Density Lipoprotein
HGC: High Gene Count
IL6: Interleukin 6
IP: Investigational Product
KEGG: Kyoto Encyclopedia of Genes and Genomes
LGC: Low Gene Count
MGS: Metagenomic Species
PP: Per Protocol
SEM: Standard Error of Mean
SD: Standard Deviation
TNF alpha: Tumor Necrosis Factor alpha
VFA: Visceral Fat Area
