# Supplementary material for: Protein supplementation during an energy-restricted diet induces visceral fat loss and gut microbiota amino acid metabolism activation: a randomized trial
Source: Sci Rep. 2021 Aug 2;11:15620. doi: 10.1038/s41598-021-94916-9 (PMC8329187; doi:10.1038/s41598-021-94916-9)
Supplement: Supplementary file 1 — Supplementary Information. [file 41598_2021_94916_MOESM1_ESM.docx]

**SUPPLEMENTARY MATERIAL**

**Protein supplementation during an energy-restricted diet induces visceral fat loss and gut microbiota amino acid metabolism activation: a randomized trial**

Pierre Bel Lassen^1,2^, Eugeni Belda^1,3^, Edi Prifti^1,3,4^, Maria Carlota Dao^1^, Florian Specque^4^, Corneliu Henegar^1^, Laure Rinaldi^5^, Xuedan Wang^6^, Sean P Kennedy^7^, Jean-Daniel Zucker^1,4^, Wim Calame^8^, Benoît Lamarche^9^, Sandrine P Claus^5^, Karine Clément^1,2,^

^1^ Sorbonne Université, Inserm, Nutrition and Obesity: systemic approaches research unit, NutriOmics, 75013, Paris, France

^2^ Assistance Publique Hôpitaux de Paris, Nutrition department, 75013 Paris, France

^3^ Integrative Phenomics, Paris, France

^4^ IRD, Sorbonne Université, UMMISCO, Unité de Modélisation Mathématique et Informatique des Systèmes Complexes, F-93143, Bondy, France

^5^ YSOPIA Bioscience, 17 place de la Bourse, 33076 Bordeaux, France

^6^ Dpt of Food and Nutritional Sciences, School of Chemistry, Food and Pharmacy, The University of Reading, RG6 6AP, Reading, UK

^7^ Department of Computational Biology, Institut Pasteur, USR 3756 CNRS, Paris F-75015, FRANCE

^8^ StatistiCal BV, Strandwal 148, 2241 MN Wassenaar, The Netherlands

^9^ Institute on Nutrition and Functional Foods, Laval University, Québec, QC Canada G1V 0A6

**SUPPLEMENTARY METHODS**

**Exclusion criteria**

Subjects were excluded if they suffered from anaemia, abnormal thyroid-stimulating hormone (TSH) level, human immunodeficiency virus (HIV) and/or hepatitis C virus (HCV) infection, severe hepatic and/or renal failure, known inflammatory disorder, diabetes mellitus. In addition, subjects receiving or having received for more than 1 week in the last 3 months drug(s) that promote visceral fat mass or inhibit its loss were also excluded (i.e. systemic steroids, neuroleptics and anti-HIV therapy). Other exclusion criteria were treatment with antibiotics within the last month, intake of food supplement or any drug known to affect body weight, satiety or appetite, previous bariatric surgery, intensive physical activity (more than 10 hours of sports activity per week), or following a hypocaloric diet or any other particular diet such as vegetarian or vegan, within the last 6 months. Subjects with serious eating disorders (anorexia, bulimia, binge disorders etc.), known food allergies (particularly to milk, gluten or soya) or a known lactose intolerance were also excluded.

**Sample size calculation**

Sample size calculation was based on the comparison of a decrease of the surface of the visceral fat mass between the two study groups using one-sided Student test and a first-degree risk alpha of 2.5% one-sided, 90% power. The number of subjects required per group was 49 (overall 98 evaluable). Using an estimated 20% dropouts, a total of 120 subjects were planned to be enrolled in the study.

**Randomization method**

Before the start of the clinical study, a randomization list was generated according to the ICH statistics guidelines (ICH Topic E 9 Statistical Principles for Clinical Trials) by the biometrics department of the Clinical Research Organization (CRO), ITEC Services. The process took into consideration the desired ratio male/female of 2/3 in each center (i.e. 20 to 28 males and 32 to 40 females per center) to ensure a balanced subgroup analysis.

**Deviations from protocol**

The protocol deviations were finalized prior to unblinding during the blind data review meeting.

Major protocol deviations were defined as follows:

- Subjects who took prohibited concomitant treatment during the study
- Non-compliance with inclusion and non-inclusion criteria
- Non-compliance with the between visits intervals as defined in the protocol
- Lack of CT-scan results
- Poor compliance (<80% IP consumption) during the first 12 weeks.

**Dates of inclusions**

A total of 376 subjects were screened for this study at two sites in two countries (France and Canada). Subjects were enrolled in the study between 30 January 2013 and 23 April 2013 in the French center and between 24 July 2013 and 15 October 2013 in the Canadian center.

**Faecal microbiota extraction, metagenomic sequencing and analysis**

*Population with faecal microbiota sampling*

All the participants of this study in the French centre had microbiota sampling before and after the intervention. A comparative table of the characteristics of the French (with microbiota sampling) vs. Canadian cohorts is shown on Supplementary Table 2.

*Extraction of faecal genomic DNA*

Participants collected faecal samples (2x1g) within 24 hours before each visit, as described in^1^. Samples were either stored immediately at −80°C or briefly conserved in home freezers, before transport to the laboratory where they were immediately frozen at −80°C following guidelines^2^. Faecal DNA was extracted using the QIAmp DNA Stool Mini kit commercialised by QiagenTM.

*Shotgun metagenomic sequencing and acquisition of gut microbiota characteristics*

DNA sequencing data were generated using Illumina HiSeq2500 sequencers. An average of 40 million reads was obtained per sample. Reads were mapped over a 9,9 million gene reference catalogue^3^. Normalization and downsizing to a sequencing depth of 15 million reads were performed using the momR package. Abundance for each MGS (metagenomic species) was computed as the mean value of the 50 most connected genes as proposed^4^. Only MGS with more than 500 genes were used in the statistical analyses. Alpha-diversity was measured in two ways: gene richness i.e. the average number of genes (meaning at least one read mapped) per sample and metagenomic species richness (MGS richness) i.e. the number of metagenomics species found in each sample. Alpha-diversity dynamic analysis were stratified on the baseline richness status defined as low gene count i.e. gene count < median of the cohort gene count (732737 genes) or high gene count i.e. gene count ≥ 732737 genes. Enterotyping of the cohort was performed following the Dirichlet Multinomial Mixture (DMM) method ^5^ using MGS abundance matrix of the entire cohort collapsed at genus level.

Functional characteristics of the metagenomes were assessed for each sample by collapsing gene abundance into KEGG orthology groups (KO groups) abundances defined as the sum of the abundances of IGC catalogue genes associated to each KO based on IGC catalogue annotation. From KO abundances, the abundances of KEGG functional modules were computed using a strategy similar to the one proposed in PMID: 22719234. Briefly, the abundance of each KEGG module is computed as the average of the upper half of its individuals KO abundances, in order to be robust to low-abundance KO groups associated to alternative enzymes. KO groups associated to more than 3 modules were excluded from module abundance computations in order to avoid the inclusion of non-specific KOs in module abundances. Only modules where at least 30% of its KO groups were present in the IGC gene catalogue were included in order to avoid the inclusion of functional modules non-related to microbiome gene functions (p.ex. eukaryotic modules). To complete this functional module matrix (where amino-acid degradation modules are not fully represented), the gut metabolic modules as described by Vieira-Silva et al^6^ were computed for each sample using GO mixer^7^.

*Projection of functions on species and constitution of amino-acid functional groups*

Using the functional annotations of the 9.9M genes catalogue, prevalence matrices (presence/absence) of functional annotation per MGS were computed. 764 KEGG modules and 29 gut metabolic modules rules were evaluated for each MGS. For a given MGS, its coverage of a module corresponds to the proportion of rules it verifies. Thus, a MGS with coverage 0.5 assumes half module's rules. Functional groups were determined by filtering MGS with a minimum coverage threshold, set to 0.5. The abundance of each functional group was computed as the sum of the abundances of the associated MGSs based on above projections and 0.5 module coverage.

To perform analysis focusing on amino acid metabolism, KEGG modules were selected using the BRITE hierarchy module. 52 modules were classified in "Amino acid metabolism" (in the "Pathway modules" upper hierarchy). Based on KEGG annotation of modules, each was assigned to biosynthesis or degradation of an amino acid. 15 module annotations were reviewed. 36 gut metabolic modules were added to complete the panel of degradation modules. For 11 of the gut metabolic modules, the rules were manually curated based on their projection on MGS. The rules of the remaining modules were literally translated from the supplementary data of Vieira-Silva S et al.^6^ For comparison purposes, same projections were carried out over a selection of 1542 reference complete genomes from KEGG database that matches with human gut prokaryotes from Magnusdottir et-al 2015 (PMID:25941533).

*16S rDNA sequencing*

After library preparation targeting the regions V3V4 of the 16S bacterial gene, 16S rDNA sequencing was performed using Illumina MiSeq 2x300 rDNA 16S technology. After demultiplexing, sequences were filtered based on quality scores (>Q30 & no N) using Prinseq v0.20.3. Paired-end sequences were matched using Flash 1.2.9 (Magoc & Salzberg, 2011), with a minimal coverage of 30 bases and 97% identity. Reads were clustered in OTUs (Operating Taxonomic Units) and further annotated phylogenetically using the Greengenes database with blastn. Abundance data (i.e. number of counts mapped) for a particular taxonomic level was normalised to the total number of good sequences per sample, and these were the values used in the following data-exploration analysis.

***In vitro* batch fermentation**

*Faecal sample collection*

Donors were recruited from the University of Reading database. They had not taken antibiotics in the last 6 months prior to the experiment and had no history of gastrointestinal disorders. Obese donors were selected with a BMI >25 and <30 kg/m2 and age > 18 and < 60 years old. Donors were instructed on how to collect fresh faecal samples in sterile plastic containers, kept in 2.5L Oxoid™ AnaeroJar™ (Oxoid, Hampshire, United Kingdom) with Oxoid™ AnaeroGen™ 2.5L Sachet (O2 <0.1%; CO2: 7-15%). These were brought to the university within 2 hours of voiding (before lunch time). Faecal samples were diluted 1 in 5 (w/v) using 1M phosphate buffered saline (PBS, Oxoid, Hampshire, UK), pH7.4. This suspension was homogenised in a stomacher (Seward, stomacher 80, Biomaster) for 120 seconds.

*Basal nutrient medium*

Basal nutrient medium was prepared with chemicals obtained from Sigma-Aldrich, Poole, UK unless otherwise stated. In one liter: 2g tryptone soy broth, 2g yeast extract (Oxoid, Hampshire, UK), 0.1g NaCl, 0.04g K_2_HPO_4_ (BDH, Poole, UK), 0.04g KH_2_PO_4_ (BDH), 0.01g MgSO_4_.7H_2_O (Fischer scientific, Loughborough, UK), 0.01g CaCl_2_.6H_2_O, 2g NaHCO_3_ (Fischer), 0.5g L–cystine HCl, 2mL tween 80, 10µL vitamin K1, 0.05g haemin, 0.05g bile salts (Oxoid), 4ml resazurin (pH7).

*Pre-digested protein mix*

A static mono compartmental digestion protocol was set up in order to reproduce the human gastrointestinal (GI) environment as closely as possible, as described in reference^8^. The three first steps of digestion were simulated (mouth, stomach and small intestine) and three fluids were prepared to mimic the physiological conditions of each step. The composition of each fluid is described in reference^8^ and pH solutions were adjusted to physiologically relevant values using 5 M NaOH and 5 M HCl solutions. The whole digestion process was performed in a 200 mL reactor controlled at 37°C under constant stirring with a magnetic stirrer over 240 min. Two grams of protein mix (powder) were added to the reactor and solubilized in 16mL salivary fluids at pH 6.8 at a final concentration of 125 g.L−1 dry matter. 24 mL of gastric fluids containing pepsin (37.5 mg) at a E:S (Enzyme/Substrate) ratio of 1:40 (w/w) were added after saliva sampling and pH solution was adjusted (2.5–3.0). After 2 h, 36 mL of intestinal fluids (24mL of duodenal juice + 12mL of bile juice) containing pancreatin (16.67 mg) at a E:S ratio of 1:50 (w/w) and 4 mL of 1 M NaHCO3 were added to the batch, pH solution was adjusted to 7 and intestinal digestion was

carried out again over two hours.

To stop the different digestion steps, every product of digestion should be filtered through a dialysis membrane (5kDa or 1 kDa). The following procedure is in use in UoR: Samples are transferred to cellulose dialysis membrane (1 KDa molecular weight cutoff) (Cheshire Biotech, Cheshire, UK) and dialysed against NaCl (0.01 M, 5°C). After 15 h the dialysis fluid is replaced with fresh fluid and dialysis continues for an additional 2 h. Samples are then frozen and freeze-dried for 5 days.

*Single stage batch culture fermentation*

Fermentation vessels with an operating volume of 20mL were set up. 18mL of basal nutrient medium was autoclaved (121°C for 15 minutes) and aseptically poured into sterile vessels. This system was left overnight with oxygen free nitrogen pumping through the medium at a rate of 2mL/min. pH meters (Electrolab pH controller, Tewksbury, UK) were connected to each vessel to regulate pH 6.7 to 6.9 with the aid of 0.5M HCl or NaOH. Each vessel was temperature controlled at 37°C and stirred using a magnetic stirrer. Pre-digested protein mix (0.35g) were added to the vessels prior to inoculation with 2mL of faecal inocula at T0. The pre-digested proteins were obtained following a gastrointestinal digestion protocol adapted from ^8^.

Samples were collected at baseline (T0) and after 48 hours fermentation (T48) and frozen at -80°C until further analysis. Faecal DNA was extracted from samples using NucleoSpin® 96 Soil (Macherey-Nagel). Bead beating was done for 1 min at 1000 g in a 1600 MiniG (SPEX SamplePrep). A minimum of one negative control was included per batch of samples from the DNA extraction and throughout the laboratory process (including sequencing). A ZymoBIOMICSTM Microbial Community Standard (Zymo Research) was also included in the analysis.

Genomic DNA was randomly sheared into fragments of approximately 350 bp. Fragmented DNA was used for library construction using NEBNext Ultra II Library Prep Kit for Illumina (New England Biolabs). Prepared DNA libraries were evaluated using Qubit 2.0 fluorometer quantitation and Agilent 2100 Bioanalyzer for the fragment size distribution. Quantitative real-time PCR (qPCR) was used to determine the concentration of the final library before sequencing. The library was sequenced using 2 x 150 bp paired-end sequencing on an Illumina HiSeq platform.

Microbiota characteristics were assessed using the same process used for the clinical study samples.

**SUPPLEMENTARY RESULTS**

**Safety analysis**

The most commonly reported individual events in both IP and CP groups were headache (N=20 and 15 patients in CP and IP populations respectively), constipation (N=15 and 18 patients in CP and IP populations respectively) and diarrhoea (N=16 and 11 patients in CP and IP populations respectively). Gastrointestinal events were the most commonly reported treatment-related TEAEs (Figure S6). Four subjects (two in each group) withdrew due to TEAEs. Severe TEAEs were scarce in both groups (N=4 and 3 patients in CP and IP respectively). There was no reporting of deaths during the study.

**SUPPLEMENTARY FIGURES**

**
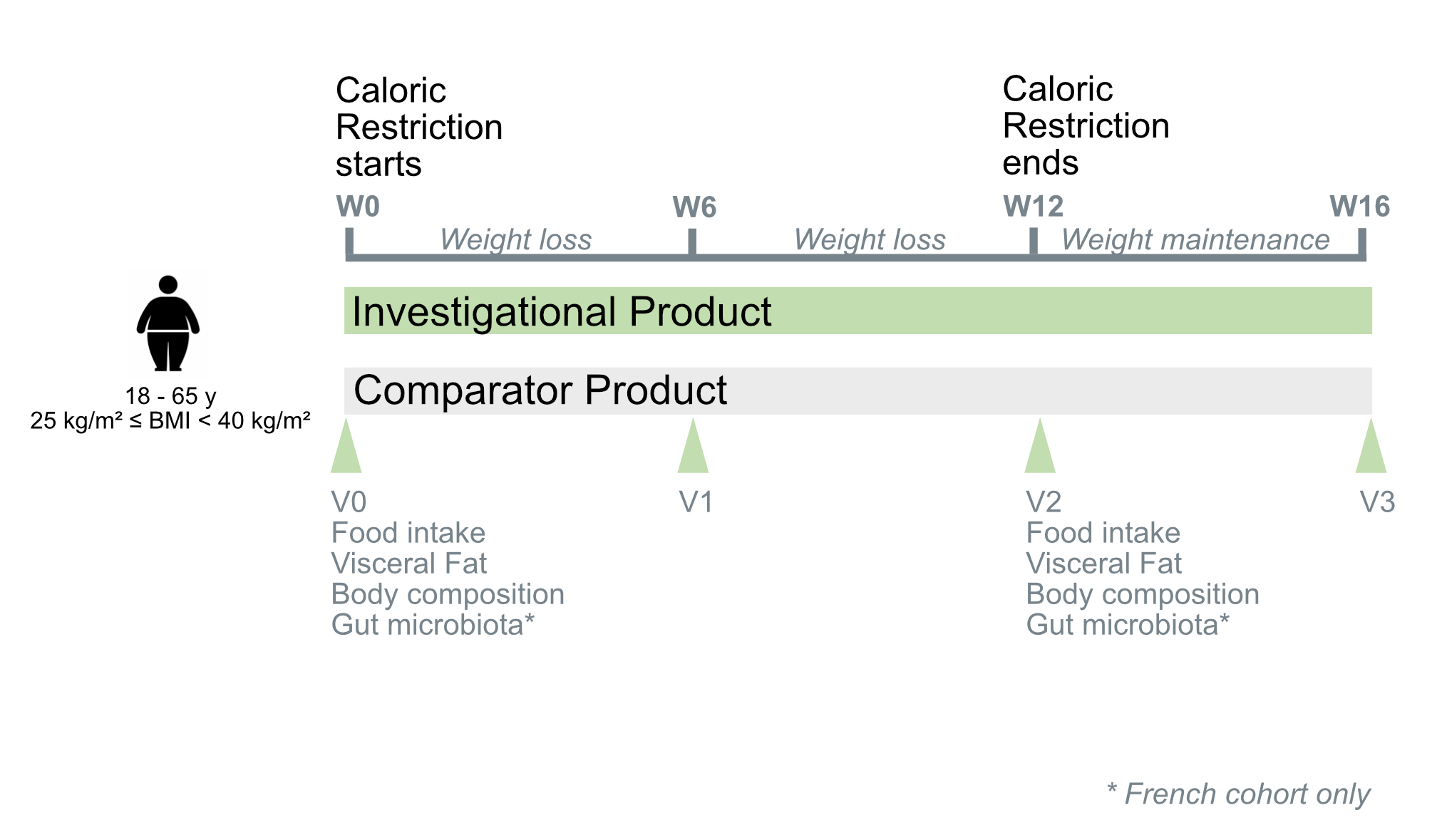
**

**Figure S1. Study Design**

CP: Comparator Product, IP: Intervention Product


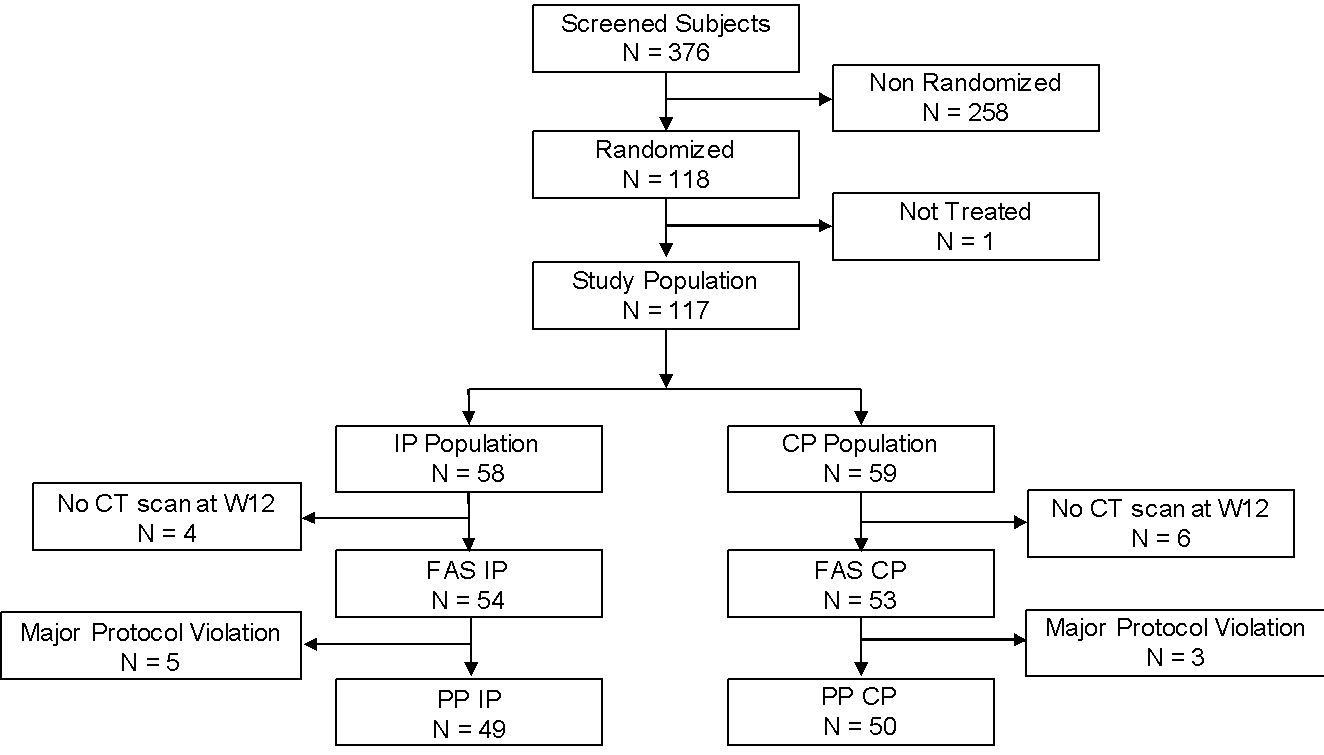


**Figure S2. Study flow chart**

W: Week, CP: Comparator Product, IP: Intervention Product, FAS: Full Analysis Set, PP: Per Protocol


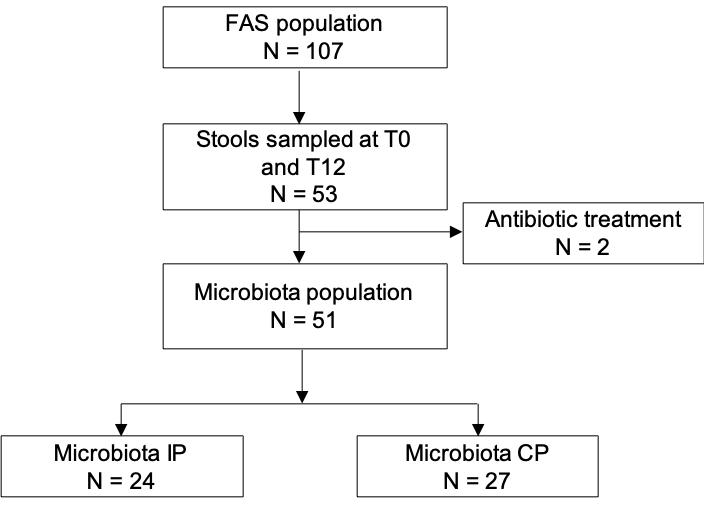


**Figure S3. Flow chart of the ancillary metagenome study. A total of 51 patients were included in the metagenome ancillary analysis.**

CP: Comparator Product, IP: Intervention Product, FAS: Full Analysis Set

**Figure S4 Effect of the dietary intervention on microbiota beta-diversity**

**A** Principal coordinates analysis (PCoA) of Bray-Curtis distance matrice of genus abundance (>20% prevalence) in all samples at baseline. Comparison of IP group vs. Comparator, permanova p=0.473. **B** PCoA of Bray-Curtis distance matrix of genus abundance (>20% prevalence) in all samples at T12 . Comparison of IP group vs. CP group, permanova p=0.343. **C** PCoA of Bray-Curtis distance matrix of genus abundance (>20% prevalence) in all CP group samples at T0 and T12. Comparison of T0 vs. T12, permanova p=0.629. **D** PCoA of Bray-Curtis distance matrix of genus abundance (>20% prevalence) in all IP group samples at T0 and T12 . Comparison of T0 vs. T12, permanova p=0.93. Figure conceived using R version 3.3.2, R Core Team (2019), https://www.R-project.org/.

**
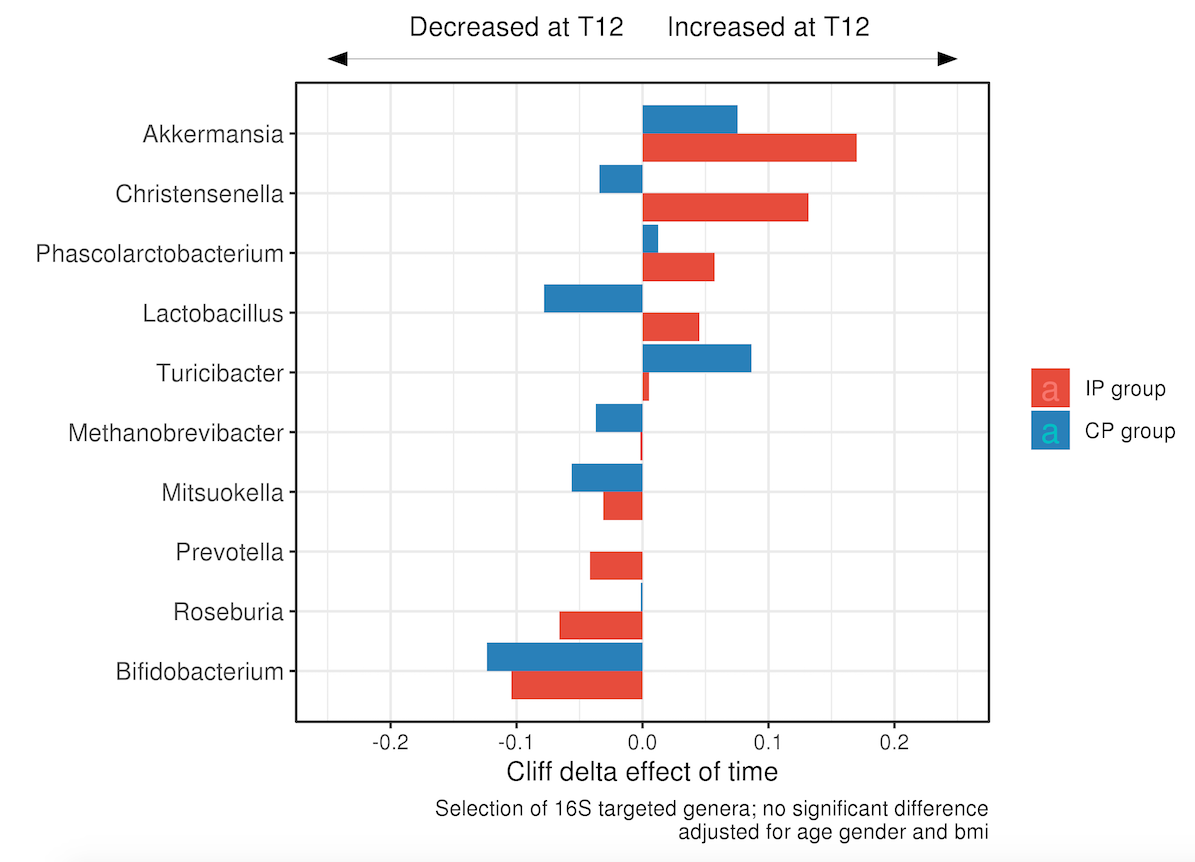
**

**Figure S5 Effect of IP on a selection of bacterial groups with known effects on metabolism (16S)**

Targeted analysis on a selection of metabolically beneficial bacterial genera measured with 16S sequencing. Bars represent the cliff delta effect of time (T12 vs T0) on genera abundance in IP group vs CP group. No significant differences are observed

IP: investigational product (high protein); CP: comparator product

Figure conceived using R version 3.3.2, R Core Team (2019), https://www.R-project.org/.

**
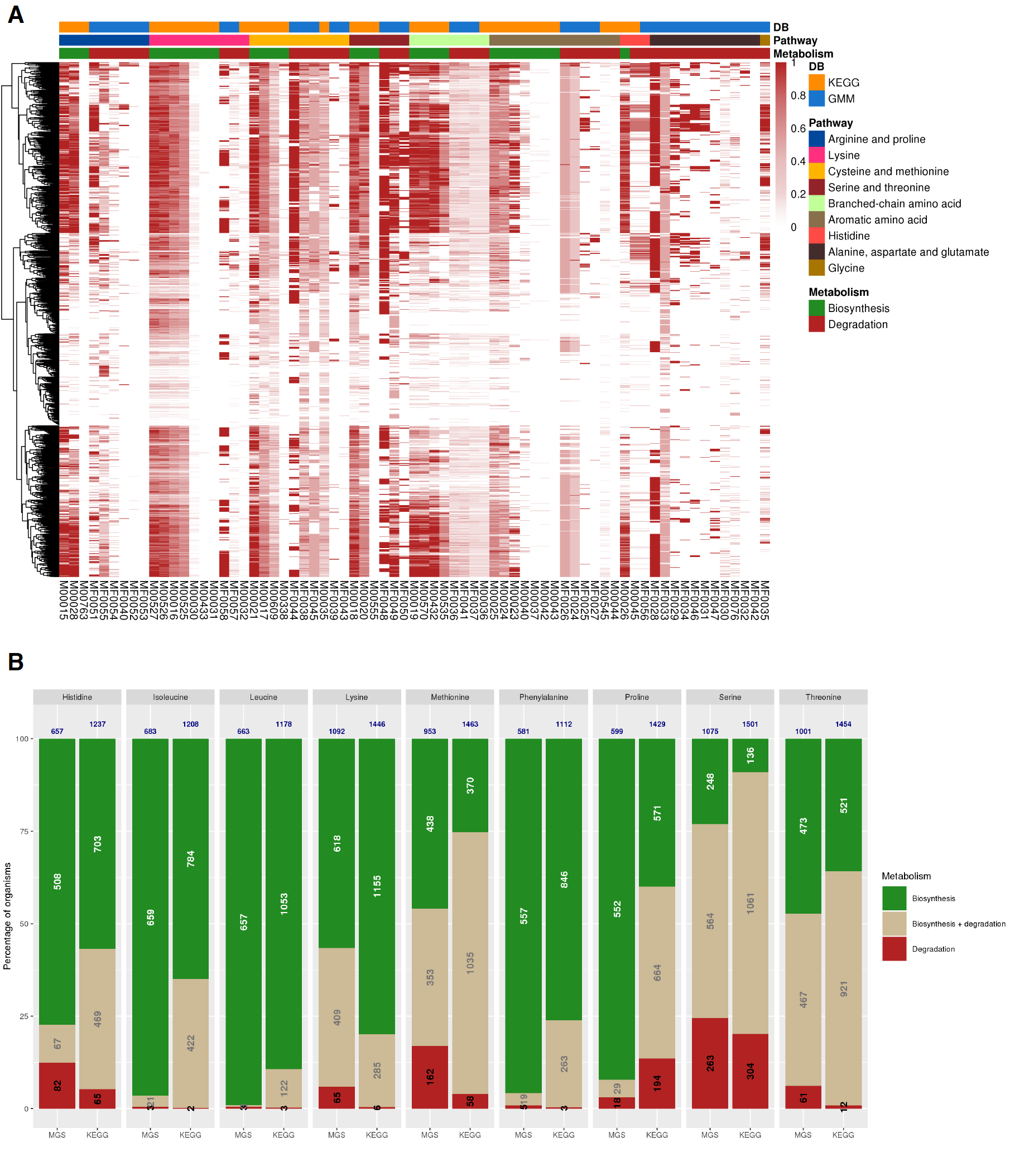
**

**Figure S6. Amino-acid metabolism modules coverage of metagenomic species**

(A) Heatmap of module coverage (from 0 to 1) of gut metabolic modules and KEGG modules related to amino acid metabolism on metagenomic species (MGSs). Coverage is defined as the number of module steps presents in the MGSs based on its gene content and KO group annotation. Rows are ordered with a dendrogram resulting of a hierarchical clustering, while columns are ordered by annotations, and mean coverage across all MGSs. (B) Comparison of the distribution of functional groups related to 9 amino acid metabolisms (those identified in the clinical study), constituted with MGSs or KEGG reference genomes (filtered to be gut-specific). Bars represent the percentage of genomes covering only degradation (red) or only biosynthesis (green) modules or both (beige). Numbers correspond to genome counts for each stacked bar (the top blue ones are the total number of genomes covering at least one module related to an amino acid metabolism). Figure conceived using R version 3.3.2, R Core Team (2019), https://www.R-project.org/.

**
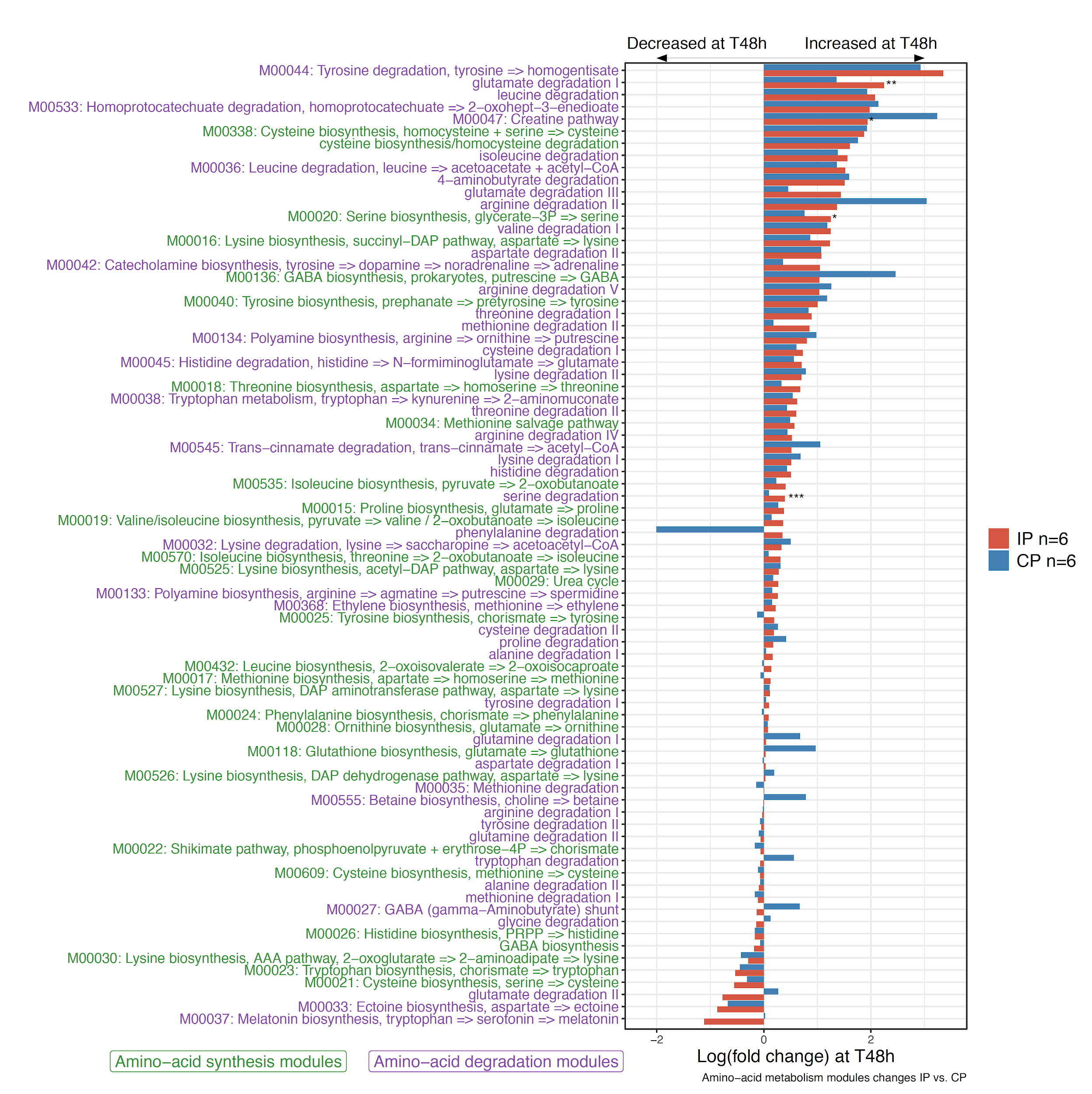
**

**Figure S7. Effects of IP on each amino-acid metabolism functional modules (48h in vitro fermentation experiment)**

Barplots representing the evolution of all amino-acid metabolism related functional modules (KEGG and GMM) following 48h of fermentation with IP or CP.. The interaction of time with investigational product (IP vs. CP), was tested in a mixed linear model with samples as random effects and baseline obesity status as fixed effects. Bars represent log(fold change) at T12 of modules abundances in each experimental condition (IP vs CP). Modules coloured in green are amino-acid synthesis modules, modules coloured in purple are amino-acid degradation modules. *: p<0.05; **: p<0.01; ***; p<0.001. No significant differences resist to adjustment for multiple comparisons.) Figure conceived using R version 3.3.2, R Core Team (2019), https://www.R-project.org/.

**
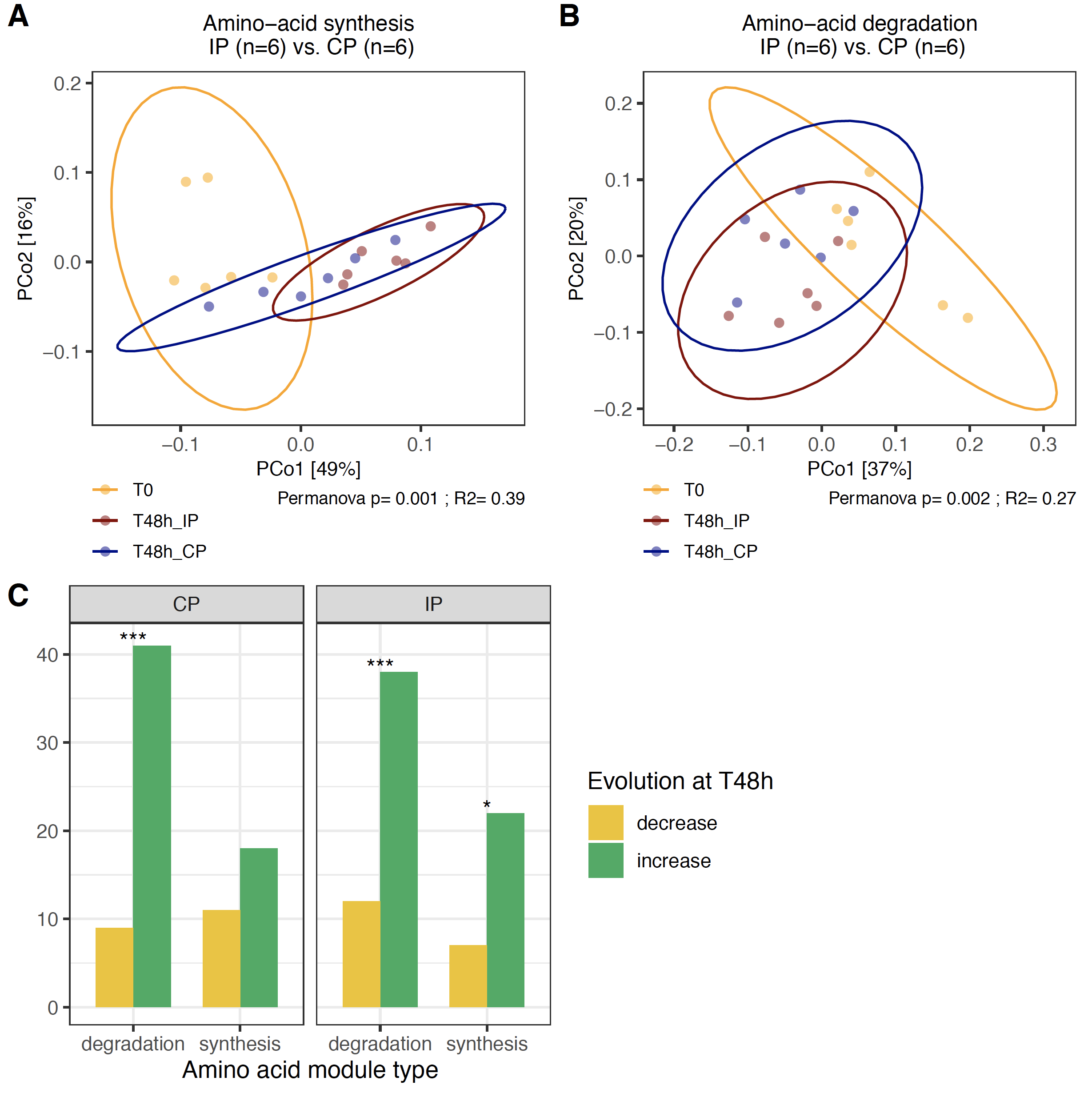
**

**Figure S8. Effects of IP on all amino-acid metabolism functional modules (48h in vitro fermentation experiment)**

Principal coordinates analysis (PCoA) of Bray-Curtis distance matrix of amino acid synthesis **(A)** and amino acid degradation **(B)** module abundance in all samples at baseline (T0) and after 48h of fermentation (T48h) with either CP or IP.

**C** Evolution of the amino acid metabolism (degradation and synthesis) functional modules (KEGG and GMM) after 48h of fermentation. Increase is defined by a mean fold change > 0 and decrease by a mean fold change < 0 for each module. The observed proportion of increased module was compared to a theoretical value of 0.5 with a binomial test. *: p<0.05; **: p<0.01; ***; p<0.001 (adjusted for multiple comparisons, FDR method). IP: investigational product (high protein); CP: comparator product. Figure conceived using R version 3.3.2, R Core Team (2019), https://www.R-project.org/.

**
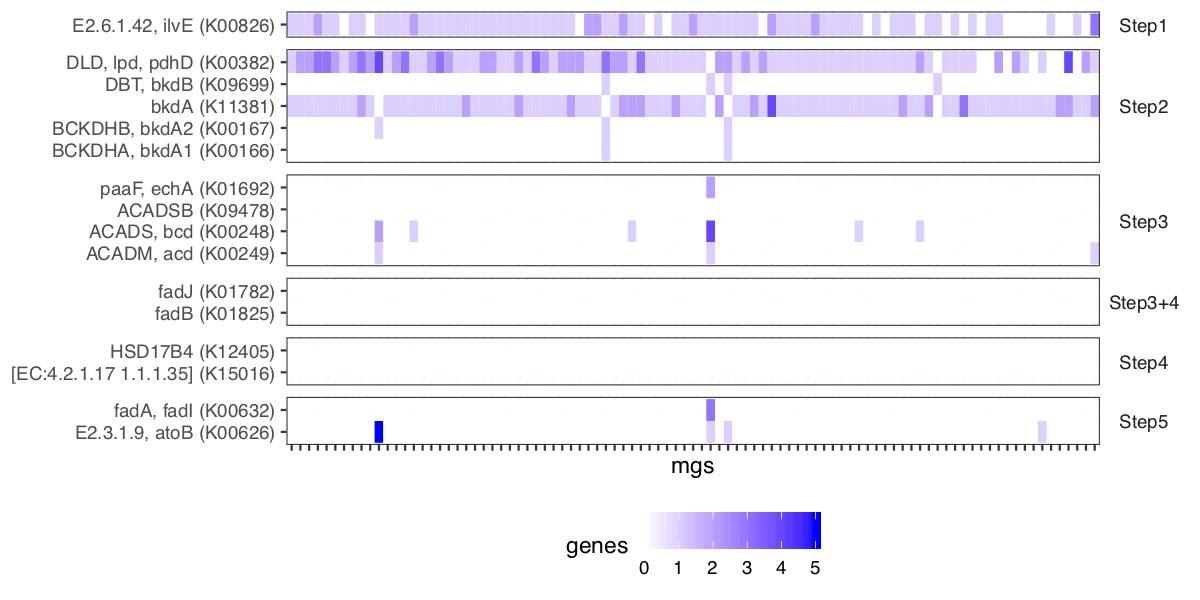
**

**Figure S9 Projection of Isoleucine degradation GMM module (MF0036) over MGSs**

Heatmap cells represents the number of genes of the different KEGG orthology groups defining the module in the MGSs (x axis). The projection is limited to MGS harbouring at least one gene of the branched-chain dehydrogenase that specifically catalyses the second step of the degradation pathway (93 MGS). Figure conceived using R version 3.3.2, R Core Team (2019), https://www.R-project.org/.

**Supplemental Table S1: Nutritional composition of the IP and CP**

|  | IP (min – max / sachet) | CP (min-max/ sachet) |
| --- | --- | --- |
| Energy (kCal) | 182 - 186 | 194 – 198 |
| Proteins (g)     Calcium caseinate (g)     Whey protein concentrate (g)     Whey protein hydrolysate (g)     Whey protein isolate (g)  Pea proteins | 33.75 – 34.02  13.53  5.76 – 6.17  5.08 – 5.48  4.2  0 | 7.30 – 7.47  3.19 – 3.50 0  0  0  3.62 – 3.88 |
| Supplemented amino acids  L-glutamine + glutamate (g)  L-leucine (g)  L-arginine (g)  Taurine (g)  L-tryptophan (g) | 7.55 – 7.66  4.39 – 4.47  1.54 – 1.55  0.36  0.86 – 0.87 |  |
| Fat (g)     Saturated fatty acids (g)     Linoleic acid (g)     α-linolenic acid (mg) | 1.46 – 2.56  0.38 – 1.03  0.594  270 | 7.56 – 7.74  1.14 – 1.48 |
| Carbohydrates (g)     Sugar (g) | 4.59 – 7.00  1.08 – 2.75 | 23.38 – 24.35  16.12 – 17.43 |
| Fibers (g) | 3.70 – 4.90 | 0.37 |
| Minerals  Sodium (mg)  Potassium (mg)  Calcium (mg)  Magnesium (mg) | 125 – 243  417 – 583  637 – 639  210 - 243 | 31  17.7  60.7 |
| Trace elements  Zinc (mg)  Chromium (μg) | 5  20 |  |
| Vitamins  Vit E (mg)  Vit B6 (mg)  Vit B9 (μg)  Vit D3 (μg) | 6  0.7  100  2.5 |  |
| Salt (g) | 0.2 – 0.3 | 0.08 |

Min composition – max or average composition is given

**Supplemental Table S2: Baseline Subject Characteristics for the PP population**

|  | **IP**  **(N=49)** | **CP**  **(N=50)** | **P value** |
| --- | --- | --- | --- |
| **Age (years)** | 47.9 (10.5) | 47.5 (11.3) | 0.83 |
| Male (N, %) | 22 (45) | 19 (38) | 0.50 |
| Female (N, %) | 27 (55) | 31 (62) | 0.30 |
| **BMI (kg/m^2^)** | 32.6 (3.9) | 32.4 (3.7) | 0.72 |
| **Waist circumference (cm)** | 105.2 (9.5) | 104.1 (8.0) | 0.56 |
| **Waist/Hip circumference ratio** | 1.0 (0.1) | 0.9 (0.1) | 0.72 |
| **Visceral fat area (cm^2^) *** | 21.9 (8769) | 18.3 (59.3) | 0.02 |
| **Fasting glucose (mmol/l)** | 5.57 (0.72) | 5.60 (0.65) | 0.90 |
| **HDL (mmol/l)** | 1.2 (0.4) | 1.2 (0.4) | 0.74 |
| **Triglycerides (mmol/l)** | 1.6 (0.9) | 1.8 (0.8) | 0.31 |

*Mean +/- SD ; BMI. Body mass index; * p<0.05 between IP and CP*

**Supplemental Table S3 Overall summary of Adverse Events in the Safety Population (N=117)**

|  | **IP**  **(N=58)** | | **CP**  **(N=59)** | | |
| --- | --- | --- | --- | --- | --- |
|  | **Patients n (%)** | **Events** | **Patients n (%)** | **Events** |  |
| **Total number of subjects with at least one event** | 53 (91.4) |  | 54 (91.5) |  |  |
| **Total number of TEAEs** |  | 194 |  | 188 |  |
| Total number of serious TEAEs |  | 0 |  | 0 |  |
| Total number of patients with TEAEs leading to withdrawal | 2 (3.5) | 2 | 2 (3.4) | 6 |  |
| Total number of patients with TEAEs leading to death |  | 0 |  | 0 |  |
| **Intensity** |  |  |  |  |  |
| Mild | 46 (79.3) | 139 | 50 (84.8) | 155 |  |
| Moderate | 26 (44.8) | 51 | 17 (28.8) | 27 |  |
| Severe | 3 (5.2) | 4 | 4 (6.8) | 6 |  |
| **Total number of TEAEs possibly related to investigational product** | 46 (79.3) | 115 | 39 (66.1) | 110 |  |

*TEAEs: Treatment Emergent Adverse Event*

**Supplemental Table S4 Summary of TEAEs Occurring in ≥5% Subjects in any Study Group by SOC (System Organ Class) in the Safety Population (N=117)**

|  | **IP**  **(N = 58)** | | **CP**  **(N = 59)** | | |
| --- | --- | --- | --- | --- | --- |
|  | **Patients,**  **n (%)** | **Events** | **Patients,**  **n (%)** | **Events** |  |
| **Immune System Disorders** | **2 (3.5)** | **2** | **3 (5.1)** | **3** |  |
| Hypersensibility | 2 (3.5) | 2 | 3 (5.1) | 3 |  |
| **Nervous System Disorders** | **18 (31.0)** | **22** | **21 (35.6)** | **27** |  |
| Headache | 15 (25.9) | 18 | 20 (33.9) | 26 |  |
| Dizziness | 2 (3.5) | 2 | 0 | 0 |  |
| **Gastrointestinal Disorders** | **45 (77.6)** | **90** | **37 (62.7)** | **80** |  |
| Constipation | 18 (31.0) | 24 | 15 (25.4) | 18 |  |
| Diarrhea | 11 (19.0) | 12 | 16 (27.1) | 21 |  |
| Abdominal distention | 5 (8.6) | 6 | 6 (10.2) | 7 |  |
| Abdominal pain | 8 (13.8) | 10 | 6 (10.2) | 6 |  |
| Gastrointestinal pain | 1 (1.7) | 1 | 2 (3.4) | 2 |  |
| Flatulence | 12 (20.7) | 12 | 7 (11.9) | 8 |  |
| Nausea | 8 (13.8) | 8 | 3 (5.1) | 3 |  |
| Gastrointestinal motility disorders | 7 (12.1) | 7 | 7 (11.9) | 7 |  |
| Vomiting | 1 (1.7) | 1 | 2 (3.4) | 2 |  |
| **Musculoskeletal and connective tissue disorders** | **7 (12.1)** | **8** | **11 (18.6)** | **14** |  |
| Arthralgia | 1 (1.7) | 1 | 2 (3.4) | 2 |  |
| Back pain | 2 (3.5) | 2 | 4 (6.8) | 5 |  |
| Ligament sprain | 0 | 0 | 2 (3.4) | 2 |  |
| Myalgia | 4 (6.9) | 5 | 2 (3.4) | 2 |  |
| **Psychiatric disorders** | **5 (8.6)** | **6** | **4 (6.8)** | **6** |  |
| Insomnia | 3 (5.2) | 3 | 3 (5.1) | 3 |  |
| **Respiratory, thoracic and mediastinal disorders** | **2 (3.5)** | **2** | **9 (15.3)** | **9** |  |
| Nasal congestion | 0 | 0 | 2 (3.4) | 2 |  |
| Oropharyngeal pain | 0 | 0 | 2 (3.4) | 2 |  |
| Cough | 0 | 0 | 2 (3.4) | 2 |  |
| **Infections and Infestations** | **24 (41.4)** | **31** | **19 (32.2)** | **22** |  |
| Bronchitis | 1 (1.7) | 1 | 2 (3.4) | 2 |  |
| Gastroenteritis viral | 4 (6.90) | 5 | 1 (1.7) | 1 |  |
| Gingivitis | 2 (3.5) | 2 | 0 | 0 |  |
| Influenza | 2 (3.5) | 2 | 1 (1.7) | 1 |  |
| Nasopharyngitis | 13 (22.4) | 14 | 12 (20.3) | 13 |  |
| Sinusitis | 1 (1.7) | 1 | 2 (3.4) | 2 |  |
| **Injury, Poisoning and Procedural Complications** | **6 (10.3)** | **6** | **1 (1.7)** | **2** |  |
| Road traffic accident | 2 (3.5) | 2 | 0 | 0 |  |
| **Metabolism and Nutrition Disorders** | **1 (1.7)** | **1** | **3 (5.1)** | **3** |  |
| Hyperglycaemia | 0 | 0 | 2 (3.4) | 2 |  |
| **General Disorders and Administration Site Conditions** | **12 (20.7)** | **13** | **10 (17.0)** | **10** |  |
| Asthenia | 1 (1.7) | 1 | 2 (3.4) | 2 |  |
| Fatigue | 8 (13.8) | 8 | 7 (11.9) | 7 |  |

**Table S5 Between-group differences in absolute changes in body composition and other metabolic variables from baseline to Week 12 in the FAS Population (N=107)**

|  | **IP**  **(N=54)** | | **CP**  **(N=53)** | | **IP vs. CP**  **(N=107)** | | |
| --- | --- | --- | --- | --- | --- | --- | --- |
|  | **Mean absolute change (SD)** | **P-value** | **Mean absolute change (SD)** | **P-value** | **Mean absolute change** | **P-value** |  |
| **Waist circumference (cm)** | -4.4 (3.6) | <0.01 | -3.6 (4.2) | <0.01 | -0.8 | ns |  |
| **Hip circumference (cm)** | -3.8 (4.0) | <0.01 | -2.7 (3.3) | <0.01 | -1.1 | 0.03 |  |
| **Waist to hip ratio** | -0.01 (0.04) | ns | -0.01 (0.03) | ns | 0.0 | ns |  |
| **Percentage fat mass (%)** | -2.2 (1.8) | <0.01 | -1.4 (2.0) | <0.01 | -0.9 | <0.01 |  |
| **Percentage lean mass (%)** | 2.1 (1.7) | <0.01 | 1.3 (2.0) | <0.01 | 0.8 | <0.01 |  |
| **Systolic BP (mmHg)** | -4.2 (11.6) | <0.01 | -5.6 (9.8) | <0.01 | 1.6 | ns |  |
| **Diastolic BP (mmHg)** | -3.5 (11.7) | <0.01 | -5.2 (9.0) | <0.01 | 1.7 | ns |  |
| **Cholesterol (mmol/l)** | -0.4 (0.7) | <0.01 | -0.4 (0.6) | <0.01 | 0.0 | ns |  |
| **Insulin (mU/L)** | -2.8 (4.2) | <0.01 | -1.9 (3.9) | <0.01 | -0.9 | ns |  |
| **HOMA-ir (UI)** | -0.7 (1.2) | <0.01 | -0.6 (1.1) | <0.01 | -0.1 | ns |  |
| **HBA1c (%)** | 0.13 (0.89) | ns | -0.04 (0.22) | ns | 0.17 | 0.03 |  |
| **Adiponectin (μg/mL)** | 0.4 (1.6) | <0.03 | 0.3 (1.8) | <0.03 | 0.1 | ns |  |
| **Leptin (ng/L)** | -5.7 (10.4) | <0.01 | -7.2 (20.4) | <0.01 | 1.5 | ns |  |
| **Creatininemia (mg/L)** | 0.5 (7.8) | ns | 1.4 (7.5) | ns | -0.9 | ns |  |
| **Urea (mmol/L)** | 1.3 (1.8) | <0.01 | 0.1 (1.1) | ns | 1.2 | <0.01 |  |

Mean values (standard deviation); Between group difference denotes IP effect versus CP. BP: Blood Pressure

**Supplemental Table S6 Between-group differences in absolute changes in body composition from baseline to Week 12 in the PP Population (N=107)**

|  | **IP**  **(N=54)** | | **CP**  **(N=53)** | | **IP Effect vs. CP**  **(N=107)** | |
| --- | --- | --- | --- | --- | --- | --- |
|  | **Mean Absolute Change (SD)** | **P-value** | **Mean Absolute Change (SD)** | **P-value** | **Mean Absolute Change** | **P-value** |
| **Visceral Fat Area (cm²)** | -20.4 (23.1) | <0.0001 | -12.6 (20.8) | 0.0001 | -7.9 | 0.014 |
| **Total Fat Area (cm²)** | -44.6 (43.3) | <0.0001 | -29.0 (43.4) | <0.0001 | -15.7 | 0.010 |
| **Subcutaneous Fat Area (cm²)** | -24.1 (32.4) | <0.000 | -16.4 (30.5) | 0.001 | 7.8 | 0.029 |
| **Fat-free mass (kg)** | -0.03 (1.22) | 0.88 | -0.63 (1.26) | 0.001 | 0.60 | 0.001 |

*Data are mean values (standard deviation); unless otherwise specified. Between group difference denotes IP effect versus comparator;*

**REFERENCES**

1. Cotillard, A. *et al.* Dietary intervention impact on gut microbial gene richness. *Nature* **500**, 585–588 (2013).

2. Thomas, V., Clark, J. & Doré, J. Fecal microbiota analysis: an overview of sample collection methods and sequencing strategies. *Future Microbiol.* **10**, 1485–1504 (2015).

3. Li, J. *et al.* An integrated catalog of reference genes in the human gut microbiome. *Nat. Biotechnol.* **32**, 834–841 (2014).

4. Nielsen, H. B. *et al.* Identification and assembly of genomes and genetic elements in complex metagenomic samples without using reference genomes. *Nat. Biotechnol.* **32**, 822–828 (2014).

5. Holmes, I., Harris, K. & Quince, C. Dirichlet multinomial mixtures: generative models for microbial metagenomics. *PloS One* **7**, e30126 (2012).

6. Vieira-Silva, S. *et al.* Species–function relationships shape ecological properties of the human gut microbiome. *Nat. Microbiol.* **1**, 16088 (2016).

7. Darzi, Y., Falony, G., Vieira-Silva, S. & Raes, J. Towards biome-specific analysis of meta-omics data. *ISME J.* **10**, 1025–1028 (2016).

8. Versantvoort, C. H. M., Oomen, A. G., Van de Kamp, E., Rompelberg, C. J. M. & Sips, A. J. A. M. Applicability of an in vitro digestion model in assessing the bioaccessibility of mycotoxins from food. *Food Chem. Toxicol. Int. J. Publ. Br. Ind. Biol. Res. Assoc.* **43**, 31–40 (2005).
